# Monoallelic Expression of Multiple Genes in the CNS

**DOI:** 10.1371/journal.pone.0001293

**Published:** 2007-12-12

**Authors:** Jinhui Wang, Zuzana Valo, David Smith, Judith Singer-Sam

**Affiliations:** 1 Division of Biology, Beckman Research Institute, City of Hope National Medical Center, Duarte, California, United States of America; 2 Division of Information Sciences, Beckman Research Institute, City of Hope National Medical Center, Duarte, California, United States of America; 3 Beckman Research Institute, City of Hope National Medical Center, Duarte, California, United States of America; Vrije Universiteit Medical Centre, Netherlands

## Abstract

The inheritance pattern of a number of major genetic disorders suggests the possible involvement of genes that are expressed from one allele and silent on the other, but such genes are difficult to detect. Since DNA methylation in regulatory regions is often a mark of gene silencing, we modified existing microarray-based assays to detect both *m*ethylated *a*nd *u*nmethylated *D*NA sequences in the same sample, a variation we term the MAUD assay. We probed a 65 Mb region of mouse Chr 7 for gene-associated sequences that show two distinct DNA methylation patterns in the mouse CNS. Selected genes were then tested for allele-specific expression in clonal neural stem cell lines derived from reciprocal F_1_ (C57BL/6×JF1) hybrid mice. In addition, using a separate approach, we directly analyzed allele-specific expression of a group of genes interspersed within clusters of *OlfR* genes, since the latter are subject to allelic exclusion. Altogether, of the 500 known genes in the chromosomal region surveyed, five show monoallelic expression, four identified by the MAUD assay (*Agc1, p* (pink-eyed dilution), *P4ha3* and *Thrsp*), and one by its proximity to *OlfR* genes (*Trim12*). *Thrsp* (thyroid hormone responsive SPOT14 homolog) is expressed in hippocampus, but the human protein homolog, S14, has also been implicated in aggressive breast cancer. Monoallelic expression of the five genes is not coordinated at a chromosome-wide level, but rather regulated at individual loci. Taken together, our results suggest that at least 1% of previously untested genes are subject to allelic exclusion, and demonstrate a dual approach to expedite their identification.

## Introduction

A number of genetic disorders are characterized by a ∼50% discordance rate between identical twins [1]. These include schizophrenia, multiple sclerosis, bipolar disorder and type 1 diabetes. While in most cases, multiple genes have been implicated in the etiology of these diseases, allelic exclusion (i.e., random monoallelic expression) of any one of the genes could account for the observed pattern of inheritance in each case. An increasing number of genes undergoing allelic exclusion are now known. The first autosomal cases were reported for T-cell receptors [2] and immunoglobulins [3]. More recently, allelic exclusion has been demonstrated for additional immune response genes [4]–[8], as well as chemosensory receptors, including olfactory receptors (*OlfR* genes) [9] and pheromone receptors [10]. A few allelically excluded genes with other functions have been reported as well [11]–[13]. Epigenetic factors contributing to monoallelic expression include DNA methylation differences at regulatory regions as well as DNA replication asynchrony; these epigenetic changes may occur early in development, providing markers for subsequent allele-specific expression (reviewed in [14] and [15]).

Because random allelic exclusion results in two simultaneous patterns of expression in a mixture of cells, it has been difficult to detect in tissues. Several groups have used allele-specific single-cell analysis [16], [17], but the method is not sensitive enough for the detection of genes expressed at low or moderate levels. An alternative approach, increasingly in use, involves detection of methylated CpG islands, which are frequently correlated with imprinting and monoallelic expression; the assays usually depend upon restriction enzyme analysis to identify differences in DNA methylation between different samples [18], [19]. The advent of high-density microarrays, including tiling arrays that contain probes for all non-repetitive sequences along a given chromosome, has led to the development of assays for detection of methylated DNA by microarray analysis [20]–[22]. We have modified that approach to detect genes that have a dual DNA methylation pattern within the same tissue. To provide proof-of-principle we chose the mouse CNS as a model system, probing the portion of mouse Chr 7 that includes the imprinted Prader-Willi/Angelman Syndrome (PWS/AS) locus [23]: The differentially methylated *Snrpn* gene lies within this locus [24], providing a positive control. Our analysis confirmed that the method can be used to identify monoallelic expression and, in addition, identifies four genes expressed monoallelically in the CNS.

To test for monoallelic expression we identified SNPs in candidate genes, and then analyzed allele-specific expression in clonal neural stem cell (NSC) lines we derived from F_1_ hybrid mice. In addition, in a separate approach, we directly analyzed allele-specific expression of genes that were selected on the basis of proximity to *OlfR* gene clusters. The rationale for selection of *OlfR* genes was that they are the major gene family thus far known to undergo random allelic exclusion in CNS tissue [9], [25], [26]. In addition, their occurrence in clusters was similar to that of most other known monoallelically expressed genes, particularly imprinted genes, that have been the most studied (http://www.mgu.har.mrc.ac.uk/research/imprinting). Using this approach, we identified an additional gene showing monoallelic expression.

## Results

### The MAUD Assay

**Figure 1:**
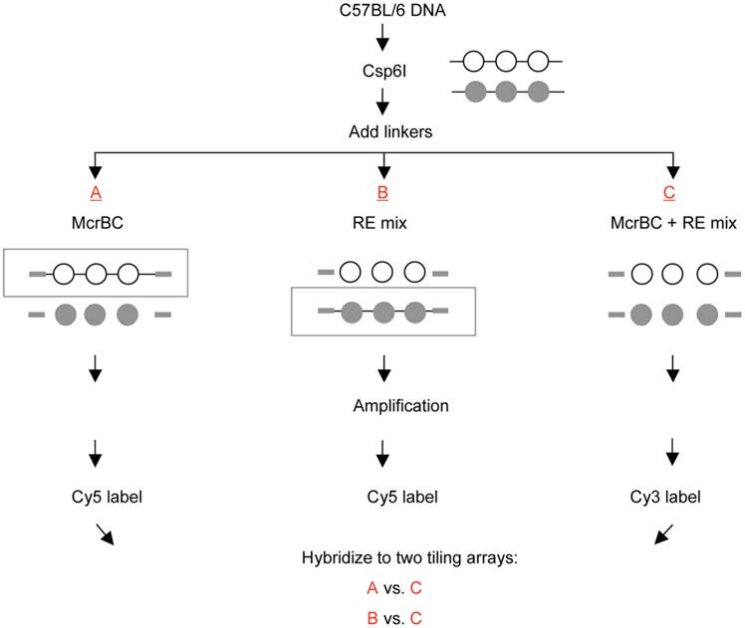
Outline of the MAUD assay. Open circles, unmethylated DNA, closed circles, methylated DNA. RE mix; AciI, HpaII and HpyCH4IV. The boxed sequences remain intact following the indicated enzymatic treatment, and are subsequently amplified by LMPCR.

Figure 1 shows a schematic diagram of the microarray-based assay that we used, modified specifically for the simultaneous detection of both methylated and unmethylated DNA sequences. Genomic DNA was cleaved with the enzyme Csp6I (G*TAC) to generate fragments ∼0.5 to 1 kb in length. Universal linkers were then added, and each sample was divided into three aliquots. The first (“A”) aliquot was digested with McrBC, which cleaves methylated DNA sequences, leaving unmethylated DNA intact. The second (“B”) aliquot was treated with a mix of methyl-sensitive restriction enzymes (“RE mix”, composed of HpaII, AciI, and HpyCH4IV) that cleave DNA only at unmethylated sites. A third (“C”) aliquot was treated with both McrBC and the RE mix, providing a negative control. Following amplification by LM-PCR, and fluorescent (Cy3 or Cy5) labeling, two microarray-based hybridizations were performed: the first compared McrBC-treated sample A with the control sample C, and the second compared RE-mix-treated sample B with the same control. Hits are defined as sequences that give a strong positive signal in both hybridizations.

**Figure 2:**
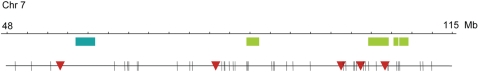
Region of mouse Chr 7 assayed and summary of results. *Upper line.* The location in Mb of the PWS/AS locus is shown by the turquoise bar, and that of the *OlfR* clusters by the green bars. *Lower line*. The location of genes assayed for allele-specific expression is shown. Monoallelically expressed genes are indicated by red triangles; bi-allelically expressed genes by vertical lines (see text).

We analyzed the 65 Mb portion of mouse Chr 7 shown in Figure 2, utilizing tiling arrays containing probes for all non-repetitive DNA at 100 bp intervals. While we examined only sequences in the vicinity of genes, we selected tiling arrays rather than promoter or GC-island arrays to include sequences that might be missed by a narrow definition of GC-rich regions or because of incomplete annotation of transcription start sites. For each gene, we scanned up to 16 kb of DNA surrounding the transcription start site(s) to include 8 kb of the 5′ region as well as the first exon and first intron.

The region we selected includes the imprinted PWS/AS locus as well as several clusters of olfactory receptor (*OlfR*) genes, as shown in Figure 2. While subsequent tests of allele-specificity necessarily relied on cells derived from F_1_ hybrids, for microarray analysis we used an inbred strain (C57BL/6), avoiding potential artifacts due to use of hybrid strains. We assayed DNA from mouse brain (cortex region, i.e., forebrain). While the forebrain is a complex mixture of various cell types, we reasoned that a high proportion of brain-derived total RNA is transcribed from neurons and astrocytes, so that a dual DNA methylation pattern in either or both of these cell types might be detectable above background.

**Figure 3:**
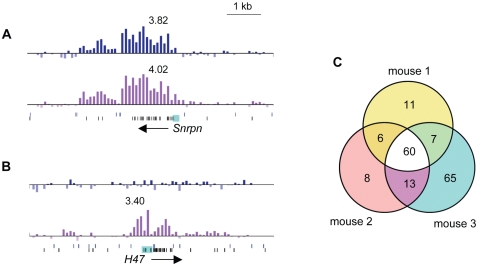
MAUD assay controls and reproducibility. Hybridization profiles are shown for A. *Snrpn* and B. *H47*. For each gene, the x-axis shows our data aligned with the nucleotide position of genes and restriction sites along mouse Chr 7 (UCSC genome browser). The y-axis represents the log_2_ ratio for RE treated-DNA vs. control (upper track, blue bars) and McrBC- treated DNA vs. control (lower track, purple bars). Each bar represents the value for an individual probe. The numbers above the peaks indicate the peak height for mouse 1; similar log_2_ ratios were found for all three mice. Below each set of tracks, the vertical lines above show the location of Csp6I sites, and the lines below show the location of DNA methylation-sensitive HpaII AciI and HpyCH4IV sites. Horizontal arrows indicate the start site and orientation of transcription for each gene. The small turquoise boxes highlight the sequences analyzed for differential DNA methylation in Figure S4. C. Evidence for reproducibility. The Venn diagram shows positive hits (genes with clear peaks in both upper and lower tracks) in the three mice assayed. Criteria for peak selection are described in Text S1.

Figures 3A and 3B show the hybridization profiles for the promoters of *Snrpn* and *H47*. While the *Snprn* gene is known to be imprinted and differentially methylated [24], *H47* is bi-allelically expressed (our unpublished data). For both genes there is a clear peak in the lower track, representing unmethylated DNA; however, only the *Snrpn* promoter shows a peak in the upper track, representing methylated DNA. Control experiments confirmed that the active and silent alleles of the *Snrpn* gene were in the McrBC- and RE mix- treated samples, respectively, and also that the *H47* promoter is unmethylated (Figure S1).

The patterns of signal intensity of the peaks in Figures 3A and 3B are compatible with the method we used to prepare the DNA samples, i.e., signal intensity was generally highest in the middle of Csp6I restriction fragments, with double peaks seen if a particular GC-rich region spanned more than one fragment. Often the central portion of a GC-rich region, containing a dense concentration of methyl-sensitive restriction sites, showed poor signal intensity, but was surrounded by sizable peak shoulders (see, for example, Figure S2, *Agc1* promoter region). Because these peaks may escape conventional peak-finding algorithms, we used visual inspection to select genes for further analysis by allele-specific RT-PCR. Of 50 genes we selected, 10 genes were not assayable for technical reasons (e.g., no SNPs or no RT-PCR product in brain). Of the remaining 40 genes, 36 showed bi-allelic expression, and four showed monoallelic expression (see below and Table S2).

As a first step in development of a computational tool for peak analysis, we used the newly found monoallelically expressed genes and *Snrpn* as a “learning tool” to optimize criteria for peak selection (Text S1). A Venn diagram (Figure 3B) summarizes the result we obtained by using the automated criteria to analyze 3 separate mouse samples. The diagram shows that the method is reproducible, and that it yields a manageable number of candidate genes that show a dual DNA methylation pattern in all three mice.

### Verification of Monoallelic Expression

**Figure 4:**
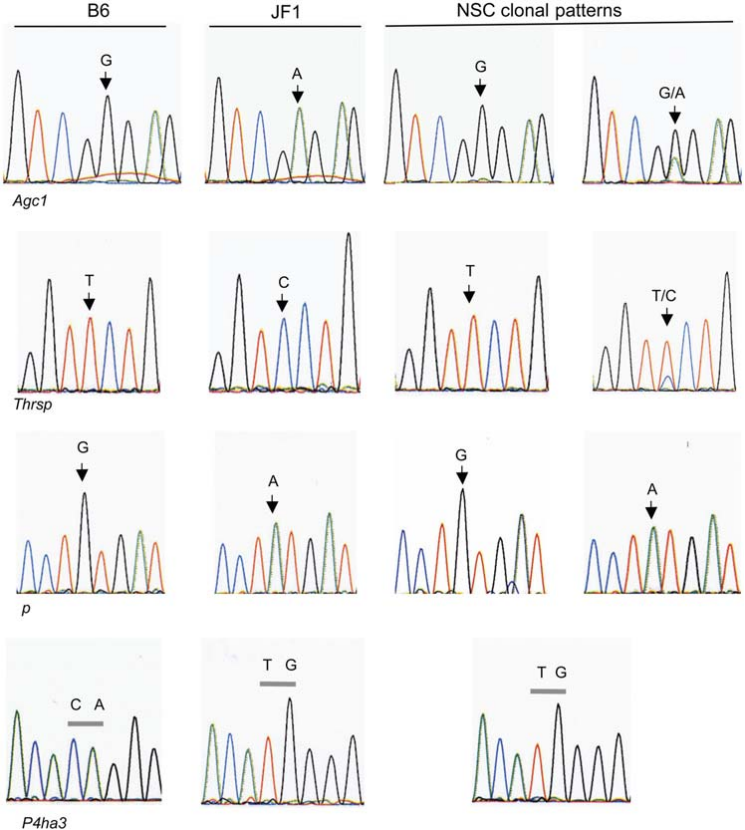
Monoallelic expression of *Agc1, p, P4ha3* and *Thrsp* in NSC clones. The panels show sequencing results following RT-PCR of RNA from representative F_1_ hybrid NSC clonal lines. Results for all six lines as well as standard curves showing the resolution of the sequencing assay are shown in Figure S3.

To test which of the identified genes showed monoallelic expression, we analyzed the RNA of six hybrid clonal lines of NSCs. Three of the clonal lines were derived from pooled CNS tissue of F_1_ (B6♀×JF1♂) female mice, and the other three lines were similarly derived from F_1_ progeny of the reciprocal cross. Figure 4 shows the different patterns of expression for the four genes found to be monoallelically expressed in at least three of the clonal lines: *Agc1*, coding for aggrecan (chondroitin sulphate proteoglycan), a necessary component of the matrix of cartilage-containing tissues; *Thrsp,* coding for a thyroid hormone- responsive protein that is expressed in the hippocampus (Allen Brain Atlas, www.brain-map.org), and that also catalyzes the synthesis of long chain fatty acids in breast tissue in human [27]; *p*, pink-eyed dilution protein (human gene *OCA2*), which is linked to albinism [28]; and *P4ha3*, coding for the alpha subunit of a protein that catalyzes the hydroxylation of proline in collagen. For two of the genes, *p* and *P4ha3*, all of the cell lines tested show monoallelic expression. The other two genes, *Agc1* and *Thrsp*, are expressed from the B6 allele in some clonal lines and bi-allelically in others. Complete results for each gene are shown in Figure S3, together with standard curves demonstrating the quantitative nature and resolution of the sequencing assay for each SNP used. For each of the genes, differential DNA methylation of a region included within the observed peaks was confirmed by bisulfite sequencing (Figure S4).

### Direct Assay of Genes near *OlfR* Clusters

**Figure 5:**
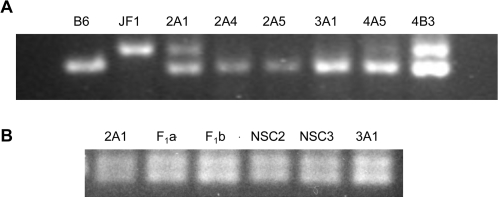
Monoallelic expression of *Trim12*. A restriction fragment length polymorphism permitted the use of agarose gel electrophoresis rather than sequencing to analyze allele-specific expression. A. RT-PCR products from parental strains B6 and JF1 and the six NSC clonal lines are shown. B. PCR of genomic DNA from two NSC clonal lines 2A1 and 3A1, the NSC parental strains NSC2 and NSC3, and brain DNA from strains F_1_ (B6♀×JF1♂) (F_1_a), and the reciprocal cross (F_1_b) are shown.

We analyzed allele-specific expression of 10 genes that are in the vicinity of *OlfR* gene clusters on Chr 7 (see Figure 2), that contain usable SNPs, and that are expressed at adequate levels in NSCs. Nine of these showed bi-allelic expression (Table S2), and one, *Trim12*, tripartite motif protein (no human homolog) showed monoallelic expression (Figure 5). In addition to its proximity to *OlfR* clusters, a BLAT search (http://genome.ucsc.edu) revealed that the 1500-bp *Trim12* gene is surrounded by five genomic sequences that are nearly identical to it (80% to 96% identity over at least 30% of the gene within a 270 kb region surrounding it).

### Summary of Results

**Figure 6:**
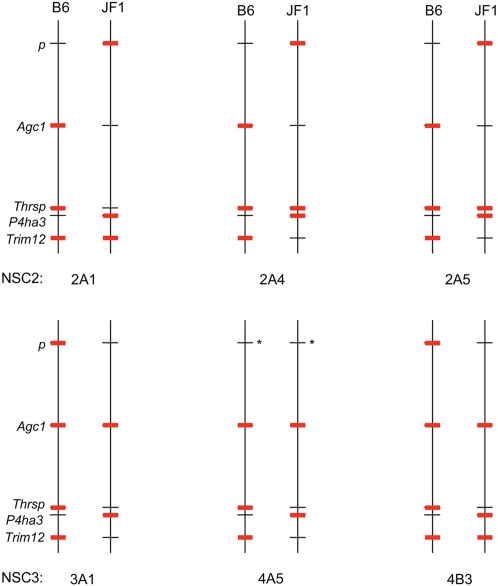
Monoallelic expression patterns are locus-specific. The region of Chr 7 from 48 Mb to 115 Mb is shown schematically (not drawn to scale). The clonal lines were derived from either NSC2 cells (JF1♀×B6♂) or NSC3 cells (B6♀×JF1♂) as shown. *, not determined.

Figure 2 summarizes our results, showing the location of the five monoallelically expresssed genes (red triangles) scattered among the 45 genes that are bi-allelically expressed (vertical lines). We see no evidence for enrichment of monoallelic expression in a particular chromosomal region. Figure 6 shows the allele-specific expression pattern of the five genes on each of the two copies of Chr 7 in each clonal NSC line. Red lines indicate the expressed allele(s) in each case. It is apparent that there is no chromosome-wide coordination of allele-specific expression. For *P4ha3*, *p* and *Agc1*, the clonal lines derived from the same cell line (i.e., NSC2 or NSC3) share the same allele-specific expression pattern. For two of the genes, *Agc1* and *p,* the parent NSC2 or NSC3 cell line is already skewed (Figure S3), suggesting that the original cell lines we obtained were oligoclonal to begin with.

For four of the genes, the results summarized in Figure 6 do not suggest any parent-of-origin effects. The results shown for *p* might suggest the possibility of imprinting, since the maternal allele is expressed in each of the 5 clonal lines assayed; however, the bi-allelic pattern seen in F_1_ (B6×JF1) brain RNA for both reciprocal crosses (Figure S3) suggests that this is not the case for most CNS cells. The possibility of imprinting in neural stem cells cannot be ruled out, however.

## Discussion

We have detected five genes that are monoallelically expressed in the CNS by analysis of DNA methylation patterns or proximity to *OlfR* genes, followed by the direct assay of F_1_ hybrid clonal cell lines. The approach should prove useful for identification of monoallelic expression in other tissues and elsewhere in the genome, the major limitation being the difficulty of obtaining clonal cell lines for some cell types.

The assay we describe for detection of methylated and unmethylated DNA has a number of advantages in addition to its potential for high-throughput analysis: Inbred strains may be assayed, avoiding artifacts due to strain differences, and the use of tiling arrays allows latitude in defining sequences of interest. However the assay also has limitations: 1) Not all allelic exclusion is associated with differential DNA methylation. An example of this is the imprinted *Ndn* gene within the PWS/AS locus, which shows heterogeneity of methylation patterns on both alleles in the brain ([29] and our unpublished data). 2) The assay depends upon having completely methylated or unmethylated DNA at the relevant restriction sites within a given Csp6I fragment: If a fragment shows partial methylation on either the silent or active allele, the signal for that allele will probably escape detection. For example, partial methylation at some methyl-sensitive restriction sites is the probable cause of our failure to observe a reproducible signal for two additional imprinted genes within the PWS/AS locus, *Peg12* and *Mkrn3* ([30] and our unpublished data). 3) Very highly GC-rich regions may show attenuated or no hybridization signal, possibly due to the difficulty of amplification. The latter two limitations are somewhat ameliorated by use of a broad window (transcription start point±8 kb); a gene is included among “hits” if any one of the Csp6I fragments within the window exceeds the signal threshold.

Although rigorous statistical analysis will not be useful until more data sets are obtained by use of our “supervised learning” approach, it seems very likely that we have enriched for genes undergoing allelic exclusion by use of our DNA methylation-based assay. Empirically we found that the assay reduced the list of potentially “interesting” genes to a manageable number (50 to 60 genes showing dual DNA methylation patterns), and that of these genes 10% were monoallelic (4 of the 40 genes for which we found usable SNPs).

We estimate that a minimum of ∼1% of previously untested genes (5 of 500 genes surveyed) undergo allelic exclusion in addition to those already known. This estimate is consistent with the findings of Gimelbrant and Chess [31], who used an approach based on asynchronous replication. For two of the genes, we found monoallelic expression in all six clonal cell lines, while for the three others, we found a mixed pattern, with some lines showing bi-allelic expression. This is not likely to be a tissue culture artifact. When brain tissue was assayed for DNA methylation, the genes showing the most clear-cut dual methylation profiles in the MAUD assay were those showing monoallelic expression in all the clonal lines (Figure S2). In addition our control experiments (unpublished) showed that the cell lines all retained silencing of X-linked genes, and a mixed pattern for genes that are mono- or bi-allelically expressed in the CNS depending upon cell type or stage of development [32].

For the five genes we report here, the pattern of allele-specific expression appears to show no chromosome-wide coordination. This was expected, based upon previously published work showing gene-to-gene variability in expression patterns, even within monoallelically expressed gene clusters. For example, cytokines and the NK cell receptors are regulated in a probabilistic manner, with cells in an activated clonal population capable of expressing either or both alleles; [4], [6], [7]. It has been suggested that this stochastic process is part of development of the immune response repertoire. IL-4, Il-5 and IL-13 lie within 140 kb of each other, and are coordinately regulated [8]; however, IL-3, which lies within 450 kb of these genes, shows allele-specific expression that is regulated independently of the neighboring cytokines.

Even when there is co-ordinated control within an imprinted domain, the regulation mechanism may involve oppositely imprinted genes within the domain. For example at the PWS/AS locus the tightly regulated paternally expressed *Snrpn* gene is within 1 Mb of the *Ube3a* gene, which is oppositely imprinted in some cells of the CNS and bi-allelically expressed in others [33]. Singh et al. [34] reported evidence for chromosome-wide co-ordination of asynchronous replication of *OlfR* and immune response genes, but their results are not necessarily at variance with locus-specific control. It may be that allele-specific expression of some subclasses of genes is regulated at a chromosomal level, or that asynchronous DNA replication of large domains does not preclude a random pattern of allele-specific expression.

*Trim12*, the monoallelically expressed gene that we selected by proximity to *OlfR* genes, is also surrounded by recently duplicated sequences and LINE elements, which are generally abundant at *OlfR* clusters [35]. Previous work has suggested a link between some monoallelic expression (and asynchronous replication) and gene duplication/repetitive elements [31], [36]. There has been recent interest in the origins of this association, including speculation that tissue-specific silencing of duplicated genes might be evolutionarily advantageous directly or indirectly [31], [37], [38]. While more work is needed in this area, it will be worthwhile to continue to use the potential link between allelic exclusion and sequence duplication in the search for new candidate genes.

### Relevance of Monoallelic Expression to Human Disorders

The search for genes undergoing allelic exclusion, in conjunction with mapping of disease susceptibility loci, may help identify candidate genes for genetic disorders that display discordance among identical twins. Such genes are also of interest in that they are more vulnerable to genetic and spontaneous mutation because allelic exclusion results in functional hemizygosity. Finally, an understanding of the silencing mechanism for genes undergoing allelic exclusion should prove valuable in designing eventual therapies in cases where such genes are implicated in specific disorders.

The potential importance of understanding allele-specific gene silencing is underscored for three of the genes we have identified, *Agc1*, *Thrsp* and *P4ha3*. Proof of monoallelic expression for these genes in human tissue would constitute a separate study, but it is worth noting that in mice, mutation of *Agc1* leads to a lethal phenotype at birth, and that there is a possible link to the human genetic disorder achondroplasia [39]. In the case of *Thrsp* and *P4ha3*, studies on humans have shown that over-expression of the genes may be deleterious, associated with a poor prognosis for breast cancer and with atherosclerosis, respectively [27], [40], [41]. For genes such as these, knowledge of the endogenous silencing mechanism might be a first step in reversing over-expression of a mutant allele or re-activating a silent one.

## Materials and Methods

### Mouse Strains and Neural Stem Cell (NSC) lines

Four-to-eight week-old C57BL6 (B6), Japanese Fancy Mouse 1 (JF1) and F_1_ hybrid female offspring of B6×JF1 mice were used. Isolation of NSC2 and NSC3 lines and the clonal cell lines derived from them is described in Text S2. Clonal cell lines 2A1, 2A4 and 2A5 were isolated from cell line NSC2, derived from F_1_ (JF1♀×B6♂) mice; clonal lines 3A1, 4A5 and 4B3 were from cell line NSC3, derived from F_1_ (B6♀×JF1♂) mice.

All clonal lines tested retained X-chromosome inactivation as measured by allele-specific expression of *Rps4x* and *Pgk1*. The chromosome number of several clonal lines tested was near-euploid, but, because of some variation in karyotype with passage number, for each gene showing monoallelic expression we verified the presence of both alleles by PCR (Figure S3).

### DNA and RNA Isolation

Genomic DNA was extracted from minced brain by treatment with proteinase K, followed by phenol-chloroform extraction and ethanol precipitation. DNA from cultured NSC lines was extracted with the Wizard® Genomic DNA Purification Kit (Promega, Madison, WI). Total RNA from brain or NSC lines was isolated by use of TRIZOL (Invitrogen, Carlsbad, CA).

### The MAUD Assay

DNA (4 µg) was digested with 20 U of Csp6I for 4 hr at 37°C (Fermentas, Hanover, MD). The resulting restriction fragments were ligated to adaptor set TA-1b as previously described [22], except that incubations were carried out at 16°C for 16 hr. DNA was then divided into aliquots, and digested in one of three ways (restriction enzymes and buffers were all from New England Biolabs (Ipswich, MA)): *A*. DNA was treated with McrBC; *B*, DNA was digested first with HpaII and HpyCH4IV in buffer 1, then with AciI in buffer 3; the two steps each consisted of two rounds of incubation, 1.5 hr each, with 3–5 U enzyme per µg DNA added at each round: *C*, control DNA was treated with McrBC and the three additional restriction enzymes. All samples were purified by use of a Qiaquick PCR purification kit (Qiagen, Valencia CA) following each enzymatic step.

Amplification by LM-PCR was carried out as previously described [22] with modification. Each ligated and digested DNA sample (600 ng) was amplified in 6 Eppendorf tubes, each containing 100 ng of DNA, 1× ThermoPol Reaction Buffer, 2.0 mM MgSO_4_, 200 µM dNTPs, 400 pm of primer TA-1b, and 3 U Vent^exo−^ (New England Biolabs), in a final volume of 100 µl. The amplified DNA was purified by use of the QIAquick PCR purification kit. Real time PCR was performed for each of the amplified DNA samples to confirm at least an 8-fold enrichment for known differentially methylated sequences in samples *A* and *B* compared with sample *C* (data not shown).

### Microarray Analysis and Data Processing

Amplified DNA samples were shipped to Nimblegen Systems Inc. (Madison, WI) for hybridization to MM8 set16 tiling arrays (mouse Chr 7 48 Mb to 115 Mb). For each mouse, results of the two hybridizations (*A* vs. *C*, and *B* vs. *C*, see Figure 1) were examined visually by use of SignalMap (Nimblegen) or the UCSC Genome Bioinformatics browser (http://genome.ucsc.edu) [42], [43]. The distance from each peak to the nearest gene on mouse Chr 7 was determined after downloading the pertinent files from the UCSC website. Peaks of interest were defined as either less than 8 kb upstream of a transcription start site or within the first exon or first intron of a given gene. “Hits” were selected for further analysis after exclusion of *OlfR* genes and genes not expressed in the brain as determined by use of the Unigene gene viewer (http://www.ncbi.nlm.nih.gov), and the Allen Brain Atlas (http://www.brain-map.org). Details of the automated peak selection protocol we used subsequently are in Text S1.

### Assay for Monoallelic Expression

Primers, SNPs and specific PCR conditions are listed in Table S1. Primers were designed by use of primer3 (http://frodo.wi.mit.edu). SNPs were identified by RT-PCR of B6 and JF1 brain RNA followed by automated DNA sequencing (City of Hope DNA sequencing Core). RT was carried out on total RNA (1.0 µg) by use of ThermoScript^TM^ kits (Invitrogen) with oligo(dT) primers. One µl of each 20-µl reaction mix was then used for amplification by PCR. Each reaction mix contained, in a final volume of 50 µl, PCR buffer II, 200 µM dNTPs, MgCl_2_, 100 pm of each primer, and 0.8 U Amplitaq DNA polymerase (Applied Biosystems, Foster City CA). After an initial denaturation (5 min 95°C), amplification was carried out for up to 35 cycles of 94°C for 45 s, annealing temperature for 45 s, 72°C for 1 min, followed by a final elongation step at 72°C for 5 min. RT-PCR products were gel-purified from a 1.5% agarose gel by use of a QIAquick Gel Extraction Kit (Qiagen) prior to sequencing.

## Supporting Information

Figure S1 DNA methylation of control genes. A. Sequence analysis of the amplified Snrpn promoter shows allele-specific DNA methylation. Two left panels: Identification of a SNP between strains B6 and JF1. Middle panel: Both alleles are present in F1 progeny of the cross (JF1MAT×B6PAT). Two right panels: The unmethylated paternal or methylated maternal allele remains intact following treatment with McrBC or the RE mix (HpaII-AciI-HpyCH4IV), respectively. The primers used were previously described to identify the same polymorphism 104 bp upstream of the major transcription start site for Snrpn in Mus musculus castaneus-Ei [1]. B. Bisulfite analysis of CpG sites within the H47 promoter. DNA from mouse forebrains was treated with McrBC or the RE mix, but only McrBC-treated DNA yielded a PCR product; amplified DNA samples were sequenced directly following bisulfite treatment without sub-cloning. Each line shows results for one mouse (n = 3). The CpG sites within the amplicon are numbered. Restriction sites for McrBC, AciI, HpaII and HpyCH4IV are indicated by M, A, H and Y, respectively. The legend to Figure S4 contains details of bisulfite sequencing. Primers and chromosomal co-ordinates for the region analyzed are listed in Table S1. Reference 1. Xin Z, Tachibana M, Guggiari M, Heard E, Shinkai Y, et al. (2003) Role of histone methyltransferase G9a in CpG methylation of the Prader-Willi syndrome imprinting center. J Biol Chem 278: 14996–15000.
(0.37 MB PDF)

Figure S2 Detection of dual DNA methylation patterns by the MAUD assay. Results are shown for genes subsequently found to be monoallelically expressed. A. Agc1. B. p (pink-eye dilution). C. P4ha3. D. Thrsp. The x-axis shows the nucleotide position along mouse Chr 7 (numbered at the top). The y-axis indicates the log2 ratio for RE mix-treated DNA vs. control (top track) and McrBC-treated DNA vs. control (bottom track). Maxium peak height (log2) ratios are indicated for peaks that are coincident in both tracks. Results are shown for mouse 1; similar ratios were found for all three mice. Below the two tracks, the blue vertical lines (top) show the location of Csp6I sites, and the black lines (bottom) show the location of DNA methylation-sensitive HpaII AciI and HpyCH4IV sites. Short horizontal arrows indicate the start site and orientation of transcription for each gene; just above each arrow, the positions of exons (bars) and introns (small arrows) are shown. The turquoise boxes highlight the regions analyzed by bisulfite sequencing (Figure S4). The figures were obtained by alignment of our custom tracks with annotation showing the location of the genes and restriction enzyme sites indicated (UCSC Genome Browser).
(0.04 MB PDF)

Figure S3 Monoallelic expression of Agc1, p, P4ha3 and Thrsp. Allele-specific expression for each gene was measured by automated sequencing of RT-PCR products containing SNPs. For A-D, Top row, brain tissue from B6 and JF1 mice and F1 hybrid progeny. Middle and bottom rows, NSC2- and NSC3- derived clonal lines, respectively. The parental cell lines NSC2 and NSC3 are shown at the left. Results of PCR of genomic DNA from representative clonal lines are shown as indicated, verifying the presence of both alleles. E. Quantitation and resolution of the assay. For each gene RT-PCR products of strains B6 and JF1 were mixed in the proportions shown prior to automated sequencing (% input). The relative intensity (peak height) of the signal for each base at SNP sites was measured to determine the % signal. For each RNA sample analyzed, if more than one base was detected at a SNP, expression was considered to be monoallelic if the predominant base comprised at least 95% of the signal. At least three technical replicates were performed for each cell line, giving the same result.
(1.31 MB PDF)

Figure S4 Bisulfite analysis of CpG sites for Agc1, p, P4ha3 and Thrsp. Upper panels, McrBC-treated DNA; lower panels, RE mix-treated DNA. CpG sites included within each amplicon are numbered. Bisulfite sequencing of DNA of mouse 1 was carried out by use of the Methylation-Gold Kit (ZYMO, Orange, CA). PCR reaction mixes contained in a 20 µl volume, 50 ng of bisulfite-modified DNA, 1× PCR buffer, 2.0 mM MgCl2, 200 µM dNTPs, 40 pm of each primer, and HotStart Taq DNA polymerase, 0.5 U (Qiagen). PCR conditions were: 95°C for 15 min, followed by 35 cycles of 94°C for 45 s, annealing temperature for 45 s, and 72°C for 40 s, followed by a final incubation at 72°C for 5 min. PCR products were purified by use of the QIAquick Gel Extraction Kit (Qiagen), and then subcloned into the vector pCR2.1 (Invitrogen) prior to sequencing. Restriction sites for McrBC, AciI, HpaII and HpyCH4IV are indicated by M, A, H and Y, respectively. Primer sequences and annealing temperatures are listed in Table S1. The chromosomal co-ordinates of the regions analyzed are listed in Table S1, and visually shown in Figure S2. Note that for Thrsp CpG site 2 appears to be methylated although it is resistant to McrBC cleavage, consistent with occasional bias that we and others observe following subcloning of bisulfite-treated DNA.
(0.05 MB PDF)

Table S1 List of primers. The list includes primers for PCR, RT-PCR and bisulfite sequencing as well as PCR conditions.
(0.03 MB PDF)

Table S2 Bi-allelic expression of genes on mouse Chr 7. The list includes 36 genes analyzed by RT-PCR of clonal hybrid neural stem cell lines
(0.09 MB PDF)

Text S1 Automated peak selection. The computational method used to analyze the peaks obtained by microarray hybridization is described.
(0.02 MB DOC)

Text S2 Isolation of NSCs and clonal cell lines
(0.04 MB DOC)
