# Supplementary figures and images for: Monoallelic Expression of Multiple Genes in the CNS
Source: PLoS One. 2007 Dec 12;2(12):e1293. doi: 10.1371/journal.pone.0001293 (PMC2100171; doi:10.1371/journal.pone.0001293)

**A**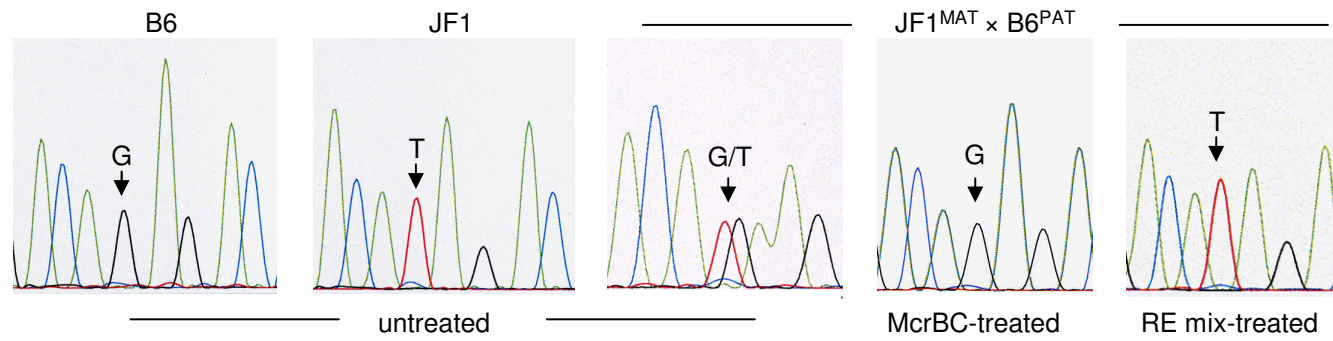**B**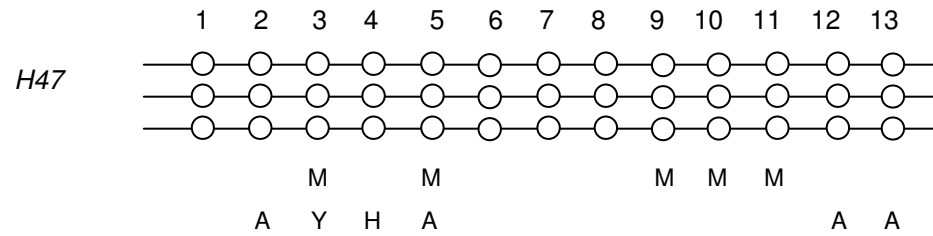

Supplement: Figure S1 — DNA methylation of control genes. A. Sequence analysis of the amplified Snrpn promoter shows allele-specific DNA methylation. Two left panels: Identification of a SNP between strains B6 and JF1. Middle panel: Both alleles are present in F1 progeny of the cross (JF1MAT×B6PAT). Two right panels: The unmethylated paternal or methylated maternal allele remains intact following treatment with McrBC or the RE mix (HpaII-AciI-HpyCH4IV), respectively. The primers used were previously described to identify the same polymorphism 104 bp upstream of the major transcription start site for Snrpn in Mus musculus castaneus-Ei [1]. B. Bisulfite analysis of CpG sites within the H47 promoter. DNA from mouse forebrains was treated with McrBC or the RE mix, but only McrBC-treated DNA yielded a PCR product; amplified DNA samples were sequenced directly following bisulfite treatment without sub-cloning. Each line shows results for one mouse (n = 3). The CpG sites within the amplicon are numbered. Restriction sites for McrBC, AciI, HpaII and HpyCH4IV are indicated by M, A, H and Y, respectively. The legend to Figure S4 contains details of bisulfite sequencing. Primers and chromosomal co-ordinates for the region analyzed are listed in Table S1. Reference 1. Xin Z, Tachibana M, Guggiari M, Heard E, Shinkai Y, et al. (2003) Role of histone methyltransferase G9a in CpG methylation of the Prader-Willi syndrome imprinting center. J Biol Chem 278: 14996–15000. (0.37 MB PDF) [file pone.0001293.s001.pdf]

A

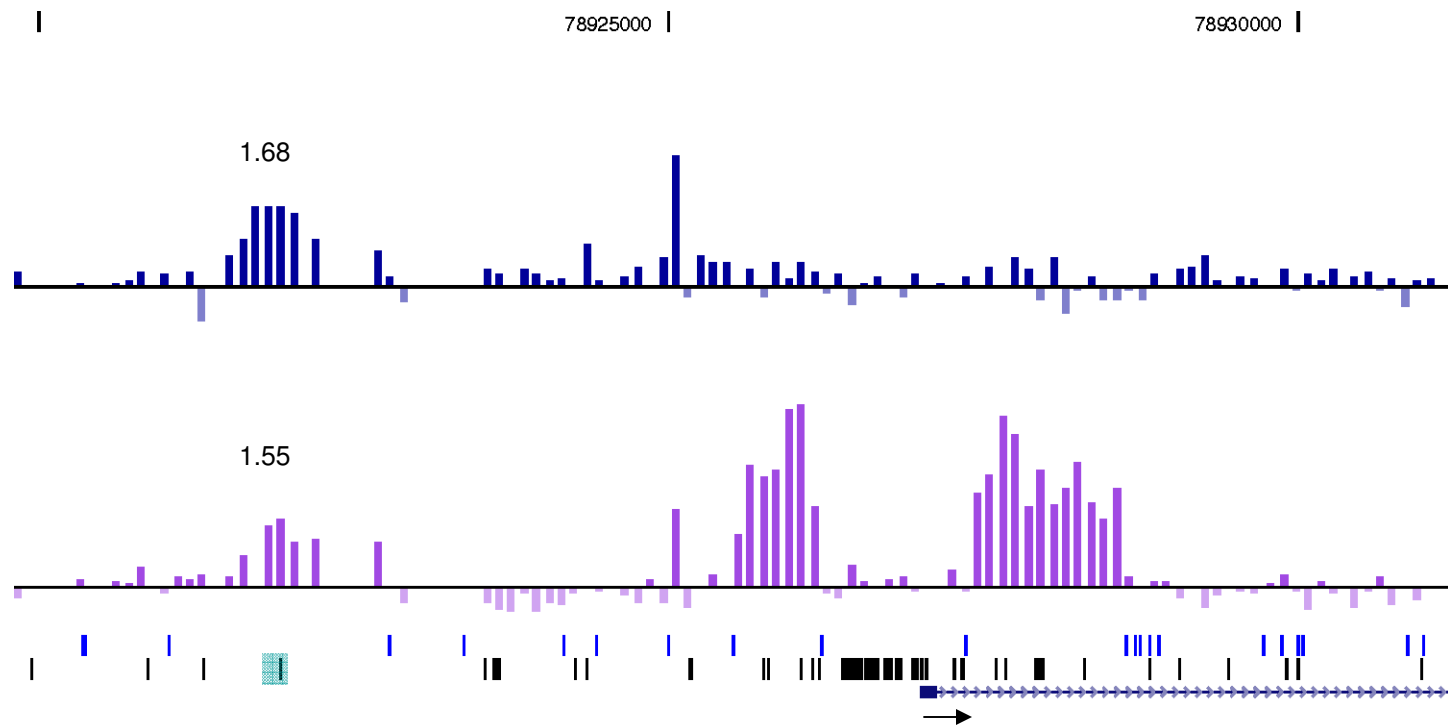

*Agc1*

**B**

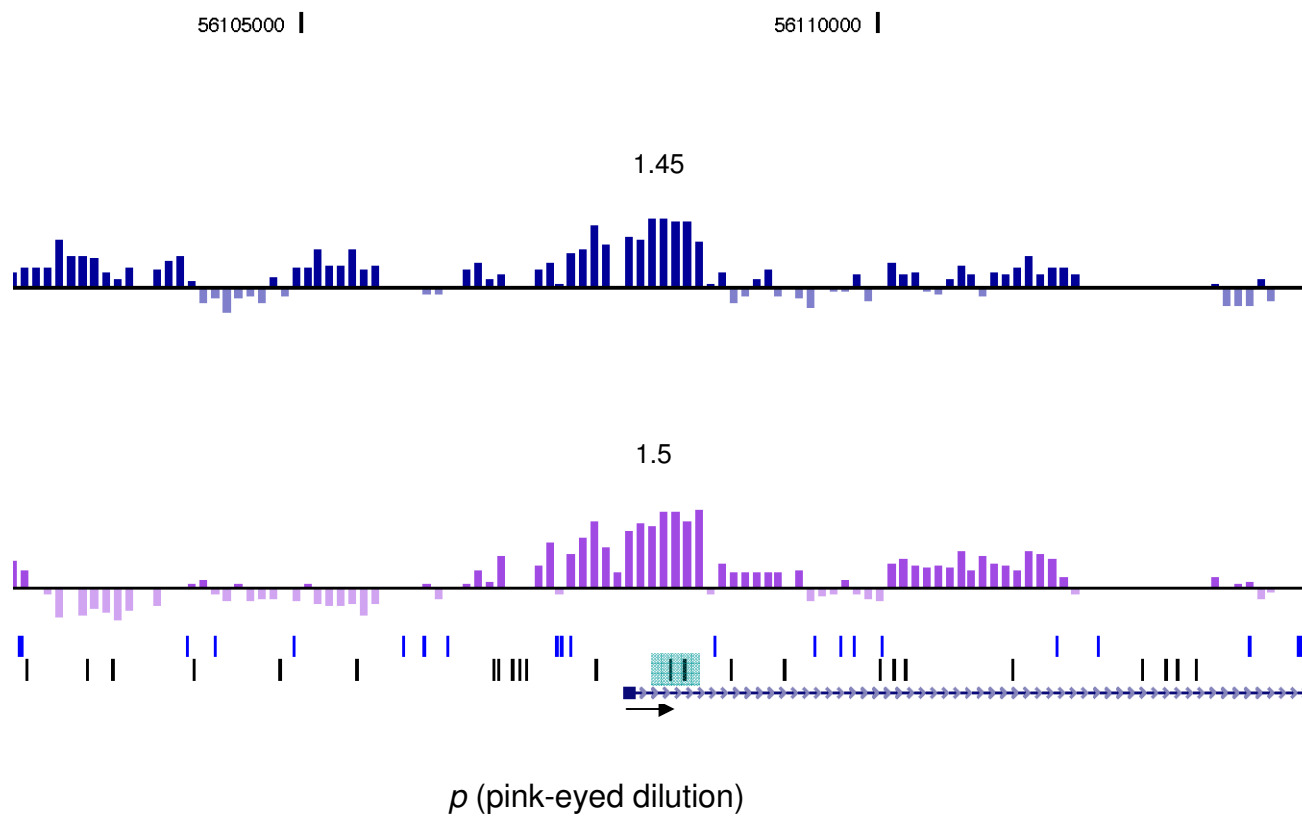

C

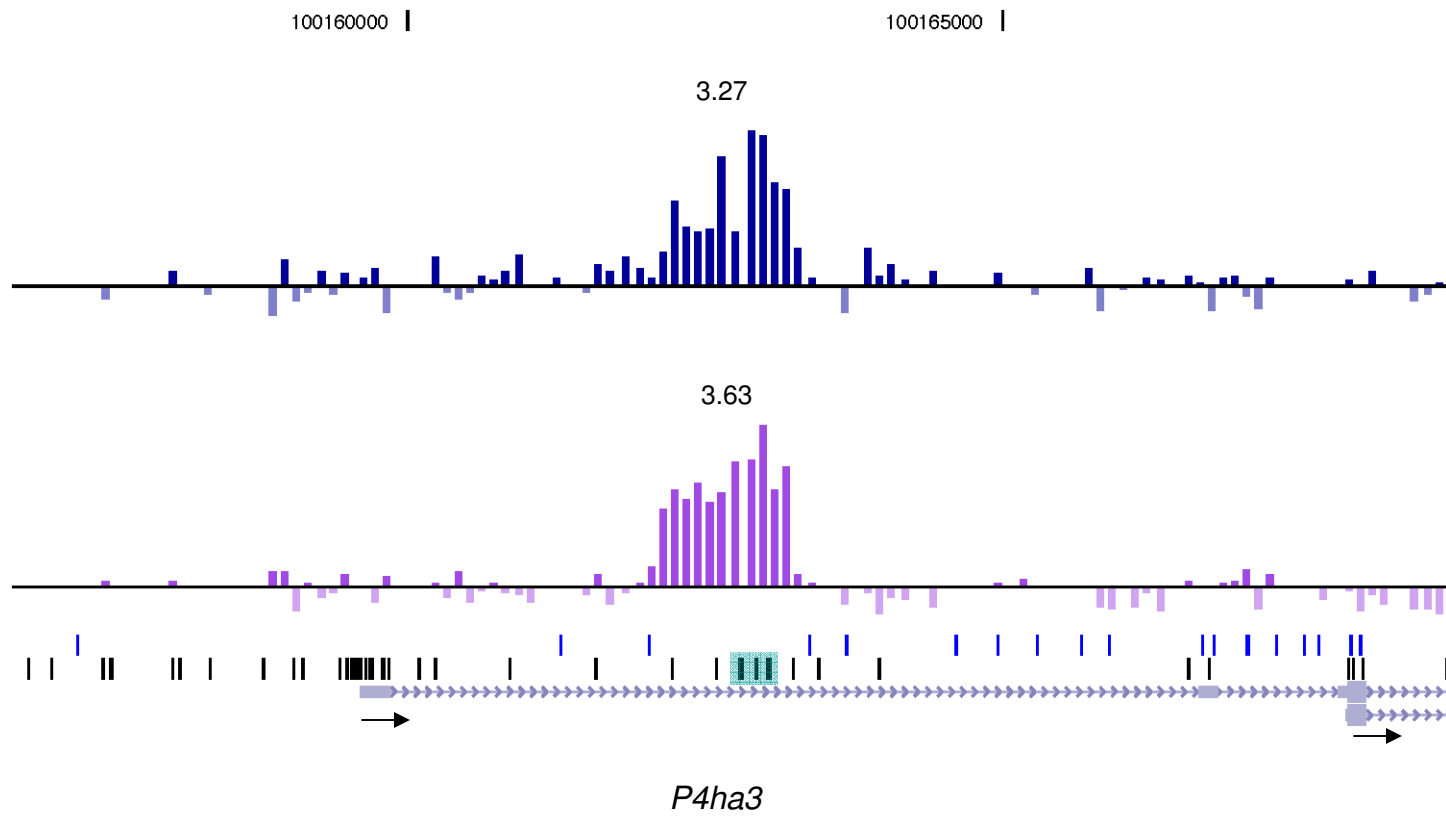

D

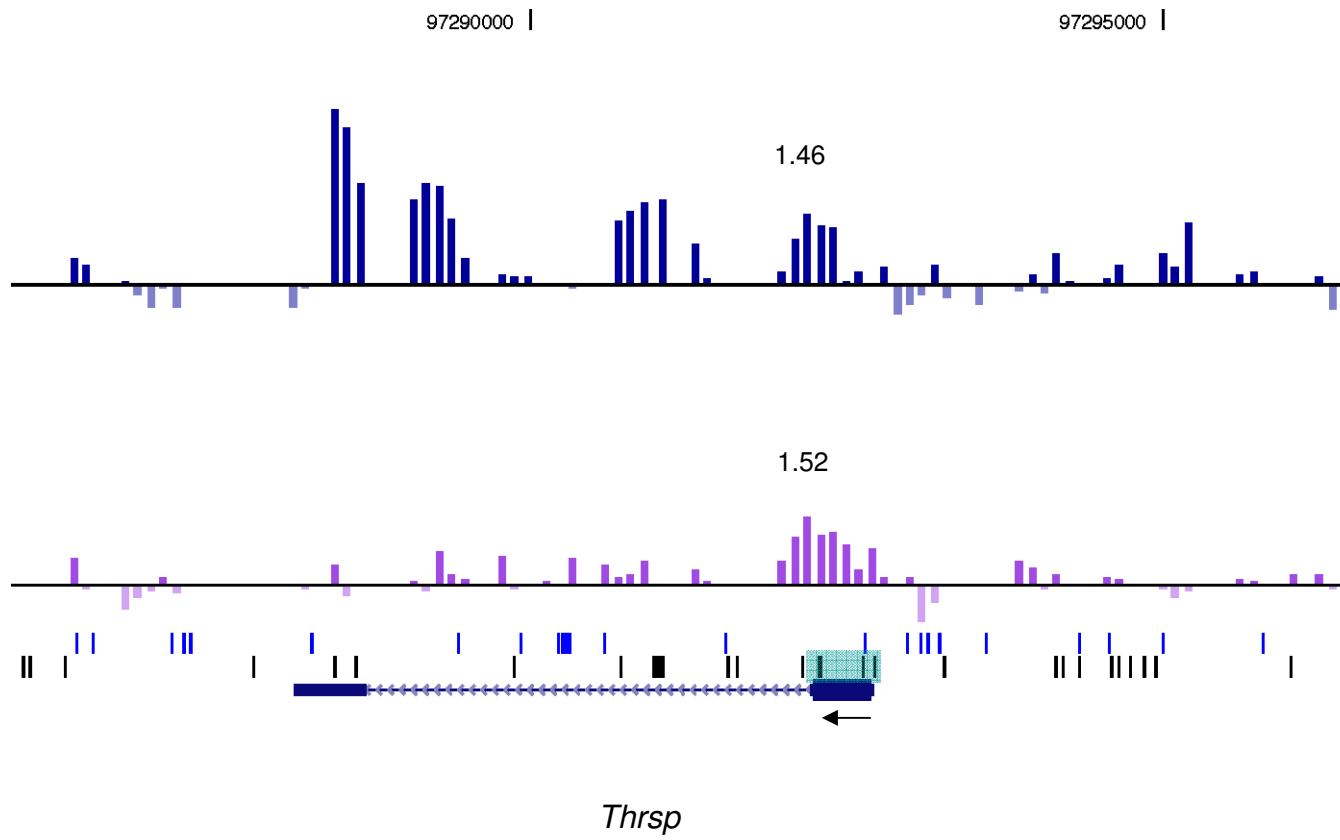

Supplement: Figure S2 — Detection of dual DNA methylation patterns by the MAUD assay. Results are shown for genes subsequently found to be monoallelically expressed. A. Agc1. B. p (pink-eye dilution). C. P4ha3. D. Thrsp. The x-axis shows the nucleotide position along mouse Chr 7 (numbered at the top). The y-axis indicates the log2 ratio for RE mix-treated DNA vs. control (top track) and McrBC-treated DNA vs. control (bottom track). Maxium peak height (log2) ratios are indicated for peaks that are coincident in both tracks. Results are shown for mouse 1; similar ratios were found for all three mice. Below the two tracks, the blue vertical lines (top) show the location of Csp6I sites, and the black lines (bottom) show the location of DNA methylation-sensitive HpaII AciI and HpyCH4IV sites. Short horizontal arrows indicate the start site and orientation of transcription for each gene; just above each arrow, the positions of exons (bars) and introns (small arrows) are shown. The turquoise boxes highlight the regions analyzed by bisulfite sequencing (Figure S4). The figures were obtained by alignment of our custom tracks with annotation showing the location of the genes and restriction enzyme sites indicated (UCSC Genome Browser). (0.04 MB PDF) [file pone.0001293.s002.pdf]

**A**

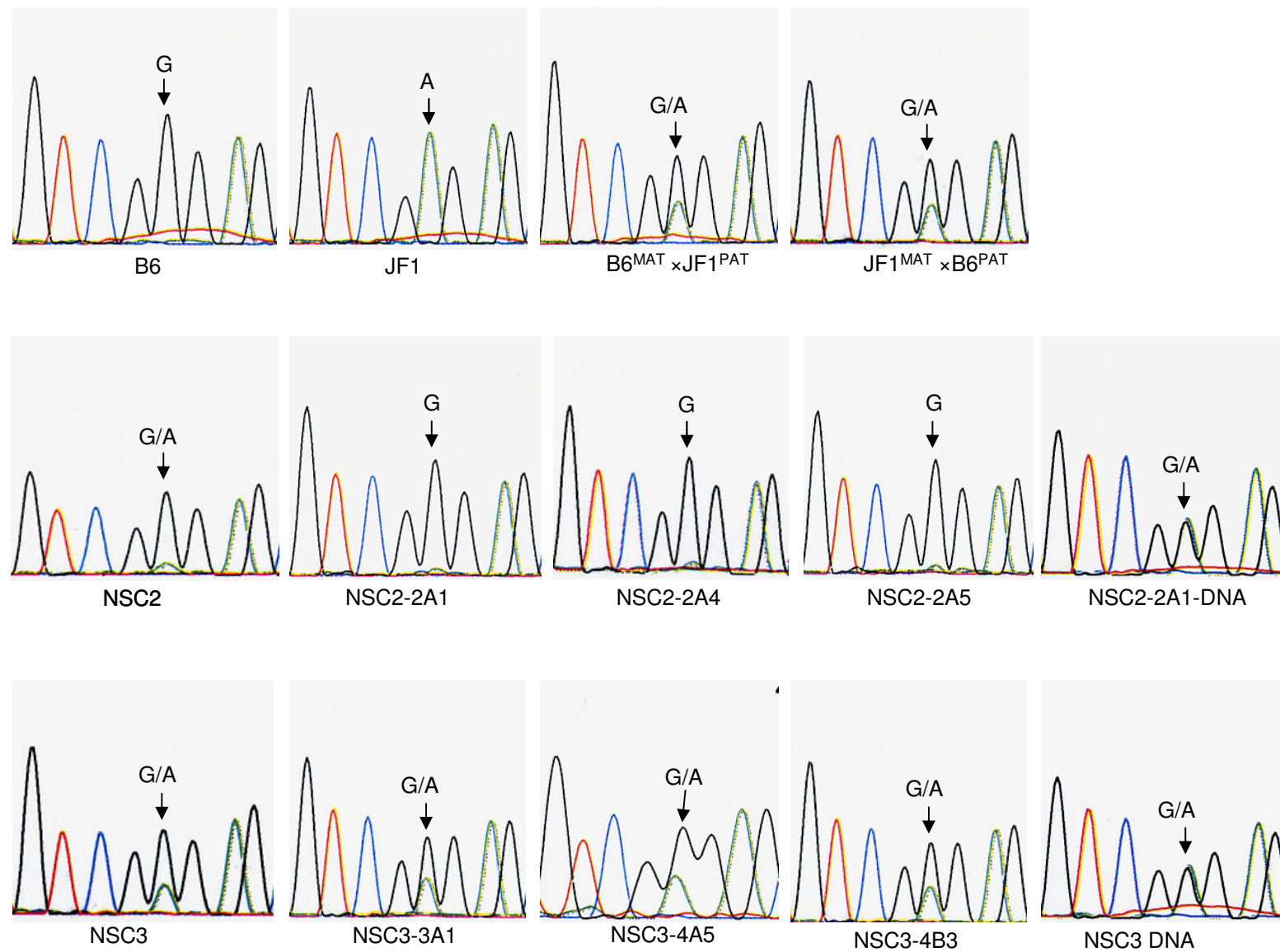

*Agc1* ( primer set 255/256)

**B**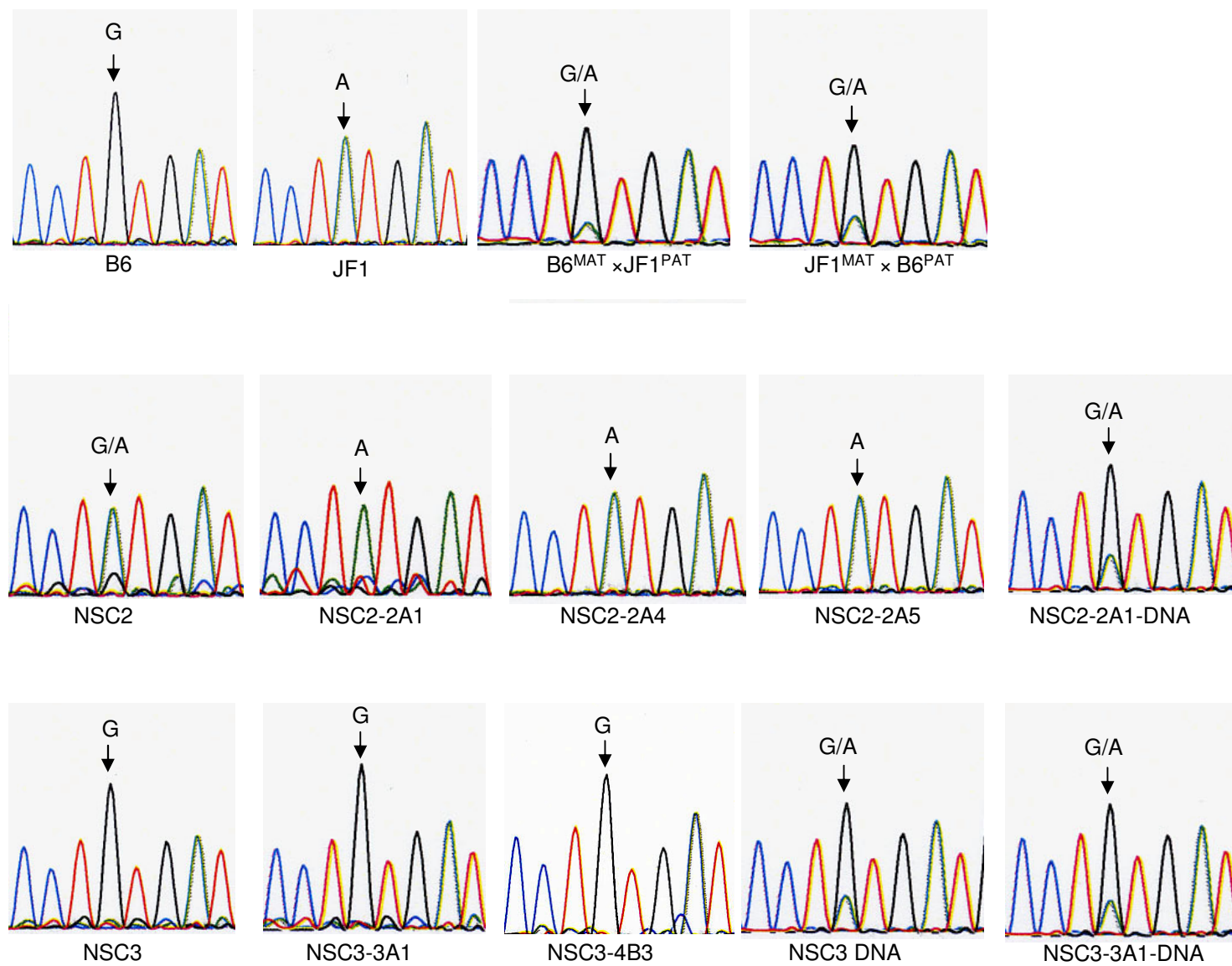

*p* (pink-eyed dilution) ( primer set 265/266)

C

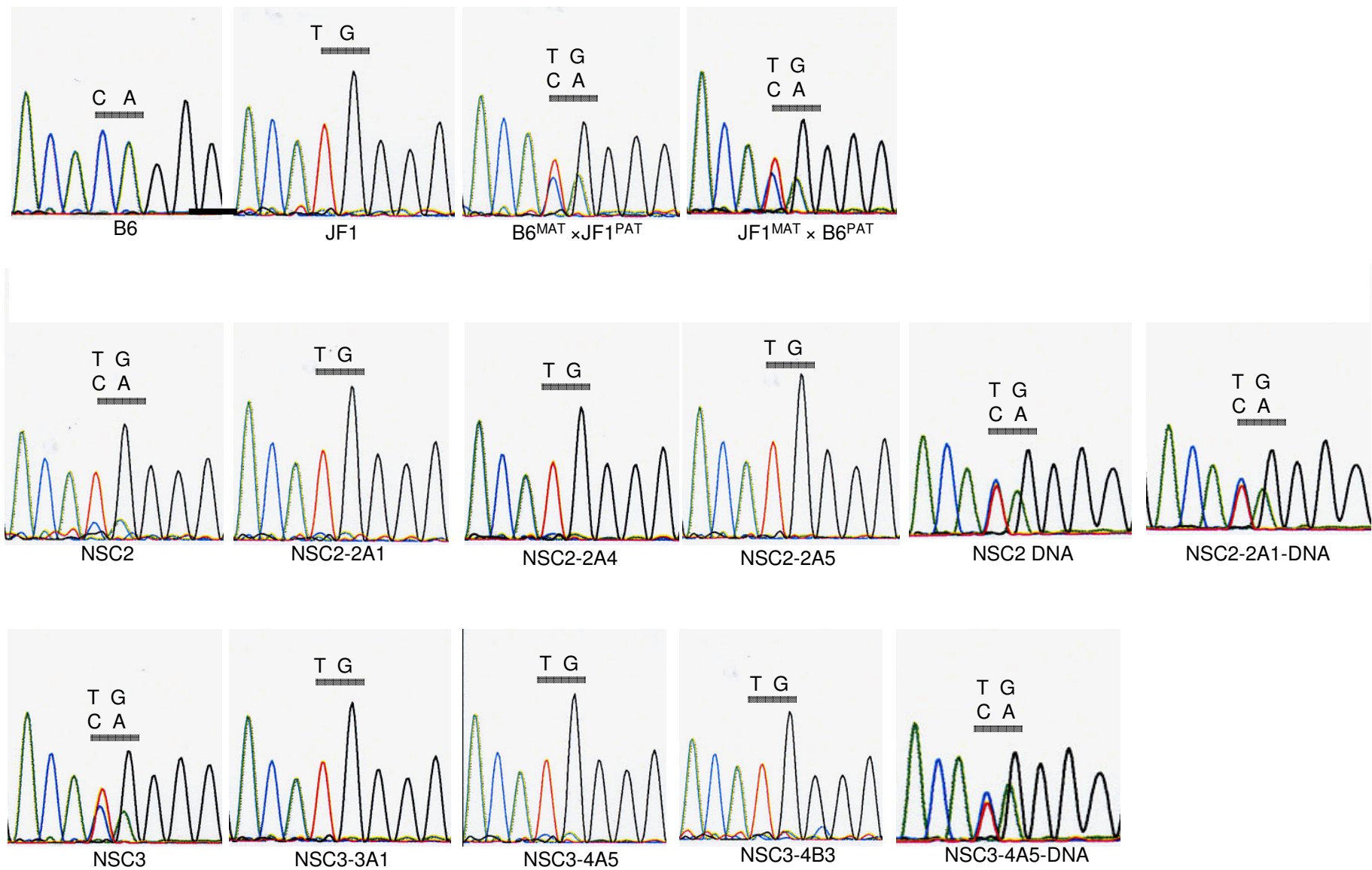

*P4ha3* ( primer set 423/424)

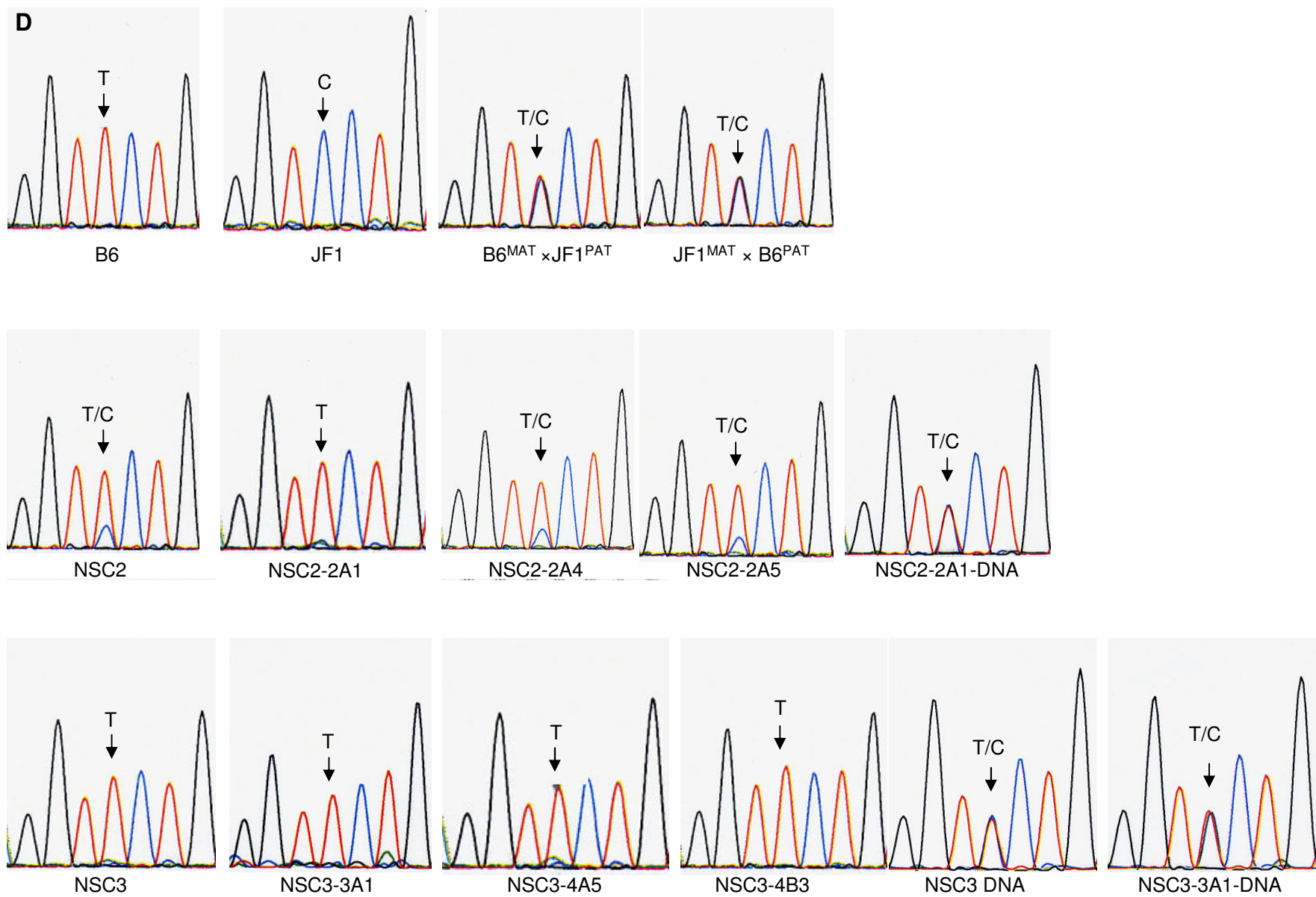

*Thrsp* ( primer set 293/294)

**E**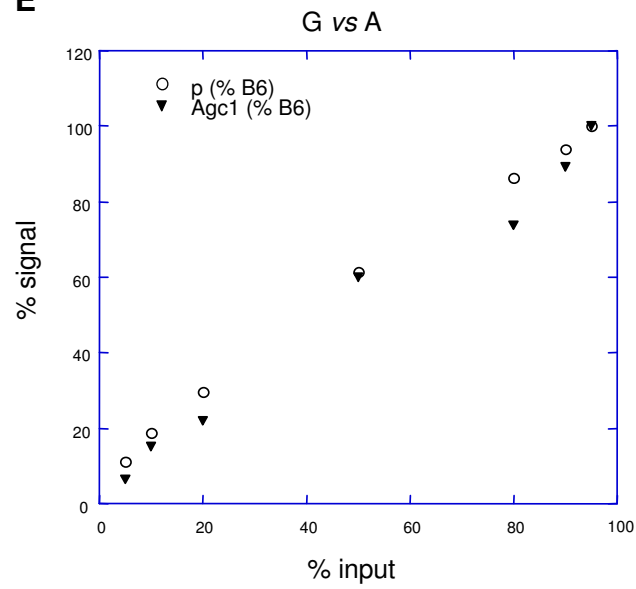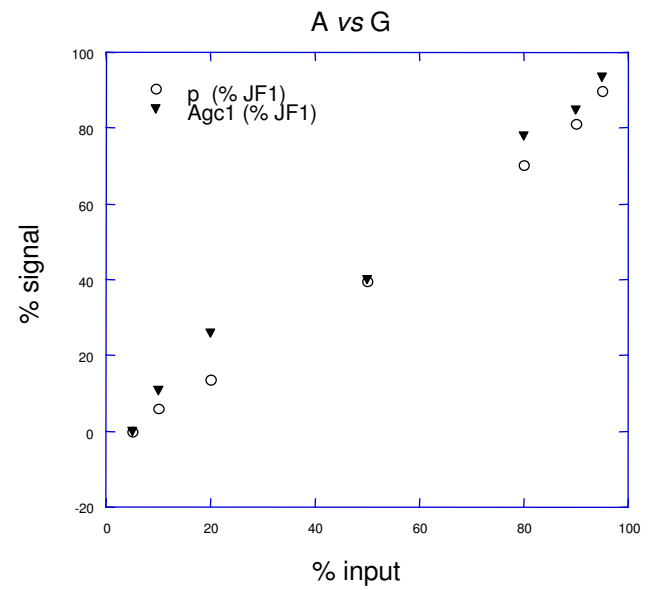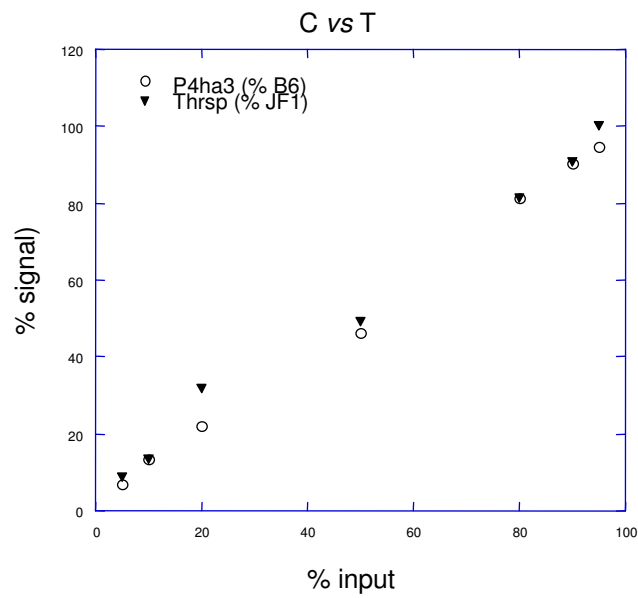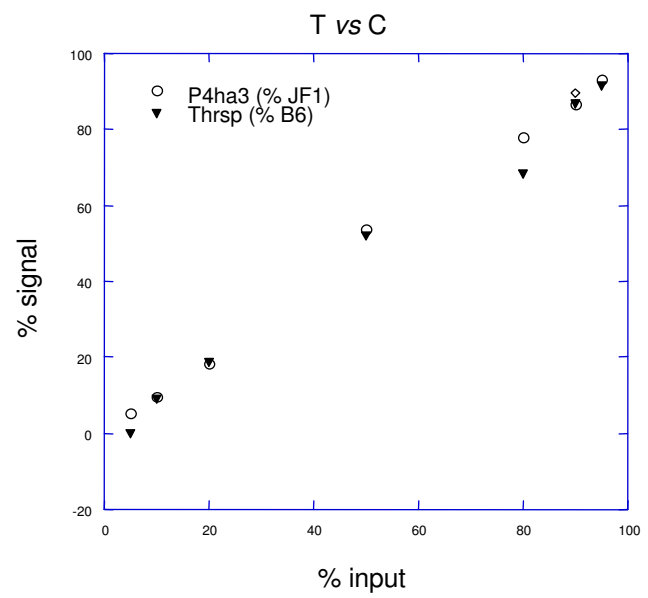

Supplement: Figure S3 — Monoallelic expression of Agc1, p, P4ha3 and Thrsp. Allele-specific expression for each gene was measured by automated sequencing of RT-PCR products containing SNPs. For A-D, Top row, brain tissue from B6 and JF1 mice and F1 hybrid progeny. Middle and bottom rows, NSC2- and NSC3- derived clonal lines, respectively. The parental cell lines NSC2 and NSC3 are shown at the left. Results of PCR of genomic DNA from representative clonal lines are shown as indicated, verifying the presence of both alleles. E. Quantitation and resolution of the assay. For each gene RT-PCR products of strains B6 and JF1 were mixed in the proportions shown prior to automated sequencing (% input). The relative intensity (peak height) of the signal for each base at SNP sites was measured to determine the % signal. For each RNA sample analyzed, if more than one base was detected at a SNP, expression was considered to be monoallelic if the predominant base comprised at least 95% of the signal. At least three technical replicates were performed for each cell line, giving the same result. (1.31 MB PDF) [file pone.0001293.s003.pdf]

**A**

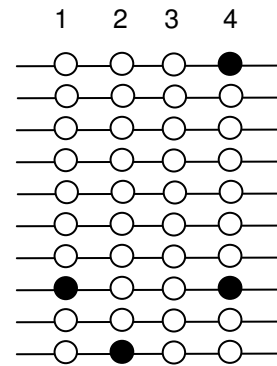

*Agc1*

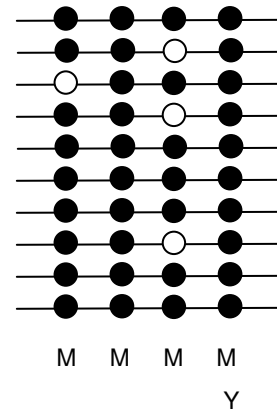

**B**

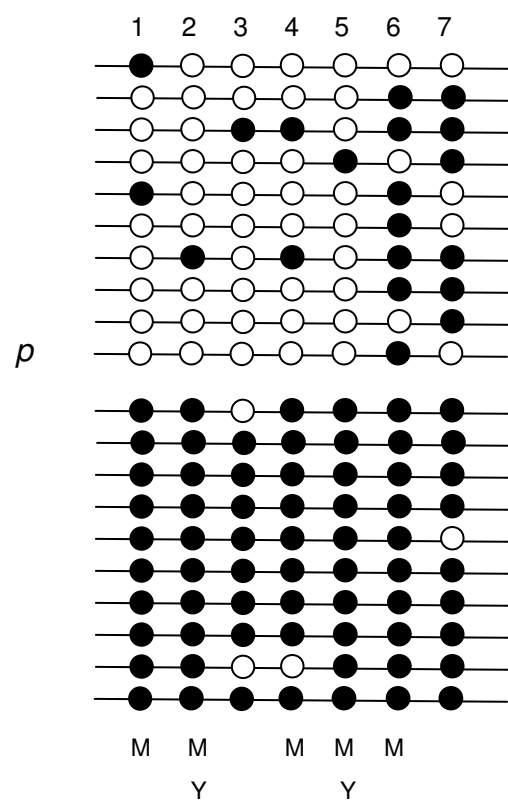

**C**

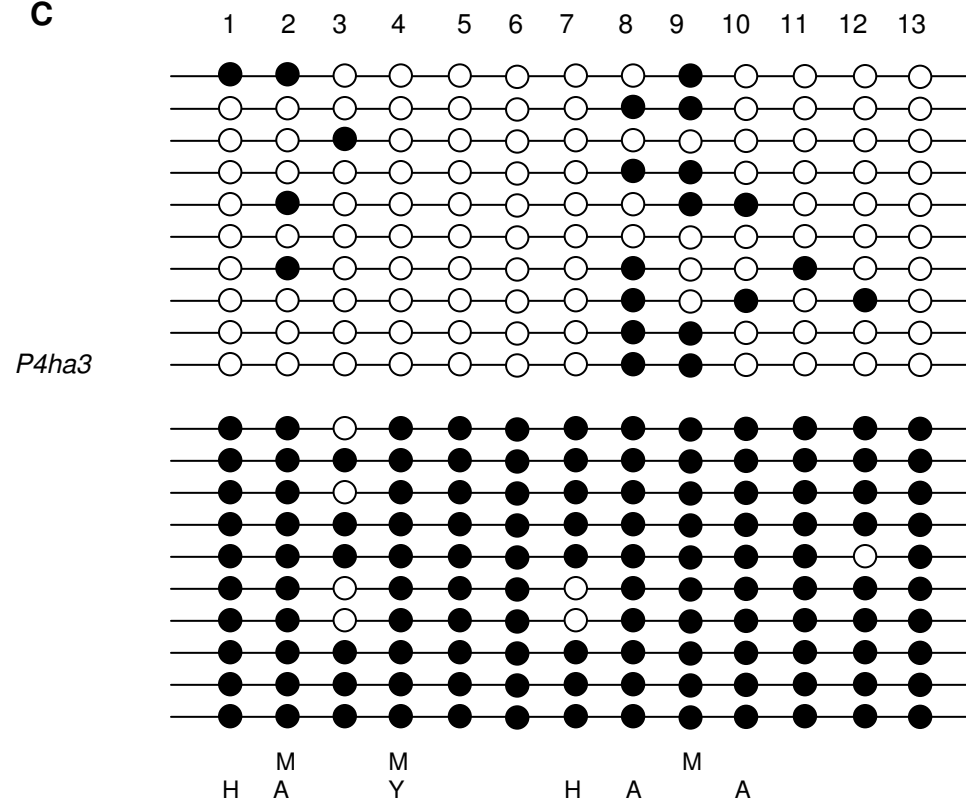

D

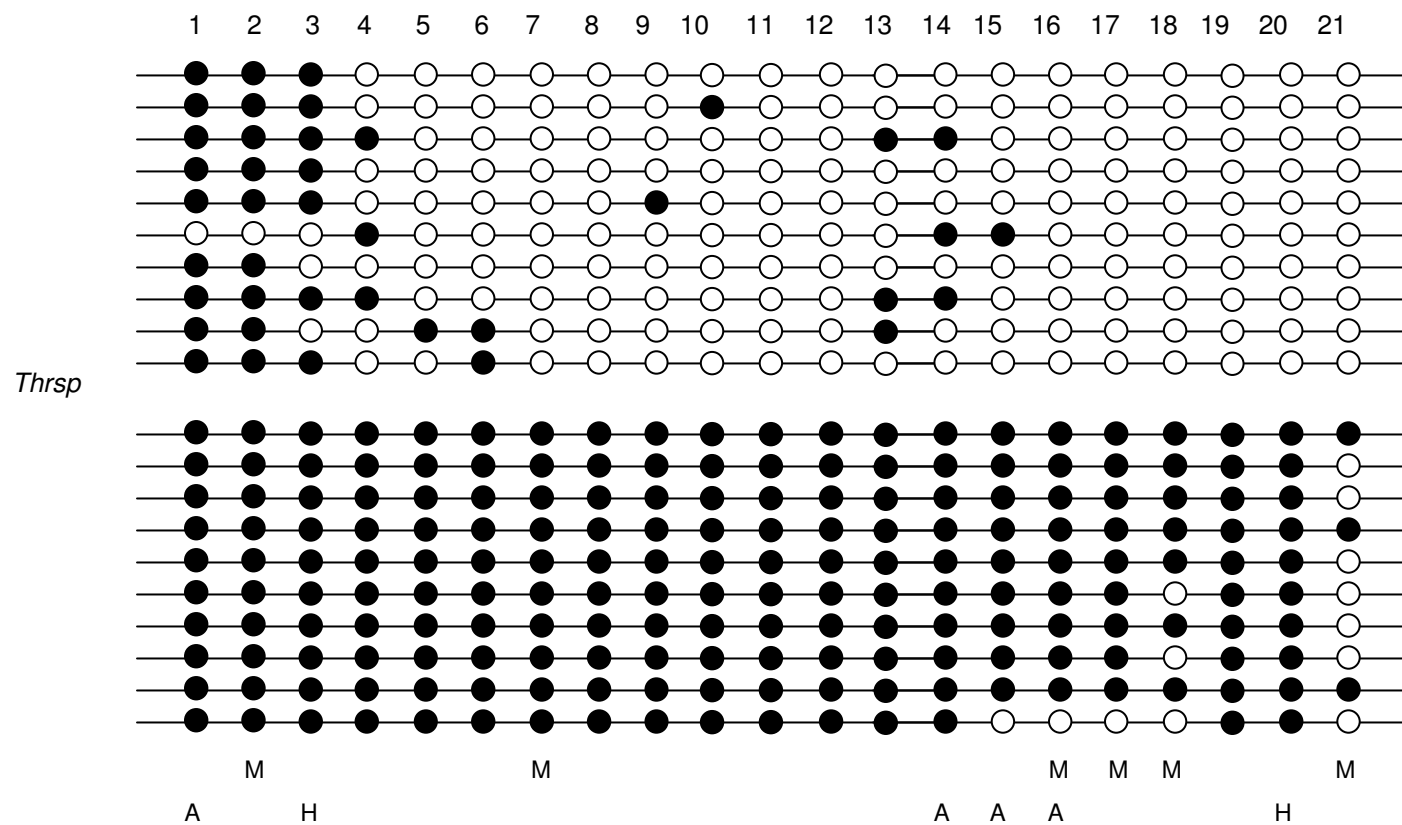

Supplement: Figure S4 — Bisulfite analysis of CpG sites for Agc1, p, P4ha3 and Thrsp. Upper panels, McrBC-treated DNA; lower panels, RE mix-treated DNA. CpG sites included within each amplicon are numbered. Bisulfite sequencing of DNA of mouse 1 was carried out by use of the Methylation-Gold Kit (ZYMO, Orange, CA). PCR reaction mixes contained in a 20 µl volume, 50 ng of bisulfite-modified DNA, 1× PCR buffer, 2.0 mM MgCl2, 200 µM dNTPs, 40 pm of each primer, and HotStart Taq DNA polymerase, 0.5 U (Qiagen). PCR conditions were: 95°C for 15 min, followed by 35 cycles of 94°C for 45 s, annealing temperature for 45 s, and 72°C for 40 s, followed by a final incubation at 72°C for 5 min. PCR products were purified by use of the QIAquick Gel Extraction Kit (Qiagen), and then subcloned into the vector pCR2.1 (Invitrogen) prior to sequencing. Restriction sites for McrBC, AciI, HpaII and HpyCH4IV are indicated by M, A, H and Y, respectively. Primer sequences and annealing temperatures are listed in Table S1. The chromosomal co-ordinates of the regions analyzed are listed in Table S1, and visually shown in Figure S2. Note that for Thrsp CpG site 2 appears to be methylated although it is resistant to McrBC cleavage, consistent with occasional bias that we and others observe following subcloning of bisulfite-treated DNA. (0.05 MB PDF) [file pone.0001293.s004.pdf]
