# Supplementary material for: Monoallelic Expression of Multiple Genes in the CNS
Source: PLoS One. 2007 Dec 12;2(12):e1293. doi: 10.1371/journal.pone.0001293 (PMC2100171; doi:10.1371/journal.pone.0001293)
Supplement: Table S1 — List of primers. The list includes primers for PCR, RT-PCR and bisulfite sequencing as well as PCR conditions. (0.03 MB PDF) [file pone.0001293.s005.pdf]

Table S1. List of primers.

Gene	GenBank No	Primer No	primer sequence	RT-PCR prod. (bp)	SNPs B6 vs JF1	SNP location* (bp)	T_A** (°C)	$MgCl_2$ (mM)
Capn5	NP_031628	227	5'-CTCCAGGCTCTGACTTCACC	451	A vs G	144	60	2
		228	5'-GCAGACGCTCAACAAAATGA					
Dbx1	NM_001005232	231	5'-CAACAGACCCACCACCTTCT	524	C vs T	193	60	2
		232	5'-AGGAGCTGGCACTCTGAAAA					
	AK158571	233	5'-ACCTGCTGATGAGTCCTGCT	476	C vs G	254	60	2
		234	5'-CCGTCAGATACCTTGGGAGA					
Tm2d3	NM_026795	237	5'-ACTGCACAGTGAAGCCCTCT	503	T vs C	250	60	2
		238	5'-CATCCATCTCCACACACAGG					
Chsy1	AK129255	239	5'-ACTGTAACCCTGGTCGATGC	503	T vs C	262	60	2
		240	5'-CAGCACAGGACAGAGAGCAG					
Lrrk1	BC072664	241	5'-CCAGGCCTCAGATGGAATTA	521	C vs T	187	60	2
		242	5'-CCCATACGATGGGATTTCAC					
Lrrc28	AK075909	243	5'-ATTGGCTTCGTGGCTTACTG	472	G vs A	110	60	2
		244	5'-AGGCAGGATCATCACCAAAC					
Akap13	AK162999	253	5'-TGAGTCAGCACCAGAAATGC	452	C vs T	349	60	2
		254	5'-CGTCACTGCCATGAGAAGAA					
Agc1	NM_007424	255	5'-GGTCACTGTTACCGCCACTT	524	G vs A	62	60	2
		256	5'-CAGGTGATTCGAGGCTCTTC					
Ap3s2	NM_009682	261	5'-ATGGTAAGTGGCAGGTGAGG	493	A vs T	114	60	2
		262	5'-CTGGGAAGCACACAGCACTA					
Luzp2	BC075646	263	5'-AATTTGTGGGCCAGAATACA	579	T vs G	274	59	2
		264	5'-TCATCCGTTGGTCAGTTTTT					
p	NM_021879	265	5'-TTGGCATCATCCTTGATTGA	622	G vs A	246	59	1.5
		266	5'-GTGGATTTGCACCATTTCTT					
Zfp592	NM_178707	273	5'-ACCGCCACCTGTTCATAGTC	631	G vs A	497	60	2
		274	5'-GTAGGAAGCCCAGAGCACAG					
Fsd2	NM_172904	275	5'-GGTTCCCGTAGGCTCACATA	648	A vs G	504	60	2
		276	5'-CAGGGTCAATACCAGGCTGT					
Tm6sf1	NM_145375	279	5'-TTTGTGATTGCCCTTTATGG	636	A vs C	277	59	1.5
		280	5'-CCTGCAATTGCTGTTTTGAA					
	AK082155	283	5'-ATGTCACCTCTCCAGGCATC	519	T vs C	317	59	1.5
		284	5'-CCTGGGCTTCTGAGTTCTTG					
	NM_029335	285	5'-GGTGCAGAACTCCATGAACA	584	C vs G	195	58	2.5

		286	5'-ATTGACCAATTGTCGGCTTC					
Fzd4	NM_008055	287	5'-AGCAGGTCACAGCTTGGAGT	451	A vs G	285	58	2.5
		288	5'-AAACAGGGGCTGCCTAGAAT					
Sytl2	AB057760	289	5'-CCCTCTCCCGTTGTCACTAA	534	G vs A	116	60	2
		290	5'-CCAGTAGGTGGCACCAAAGT					
	BC066148	291	5'-GGCTTAAATGGTGCTTCCAA	613	T vs A	90	59	1.5
		292	5'-TCCAAACAGATCTGCCATCA					
Thrsp	NM_009381	293	5'-CCGAAGAAGACAGGATCTCG	626	T vs C	142	60	2
		294	5'-CTTTCCCCAGGGATAAAAGC					
aquaporin 11	NM_175105	295	5'-CCCACCTGGACTCTGACACT	612	A vs C	522	60	2
		296	5'-TCGAGTCTTTGGGAGTGGTT					
Gdpd5	NM_201352	301	5'-GTGCTCCAAGCTCCTTTGTC	627	C vs T	143	58	2
		302	5'-GGGAACCTGAGGTCAGATCA					
Plekhb1	BC024756	309	5'-TTCTCCCCATTAAGCCCTCT	609	G vs A	143	60	2
		310	5'-GGCATTTCCTAATGGAAGCA					
P2ry2	NM-183168	311	5'-ACAGAGGCTCACAGCCAAGT	538	C vs T	394	59	1.5
		312	5'-TGCAAAAGTCTTGGCAAATG					
Pde2a	AK159012	315	5'-AGGCCTCCCCAGTAACAACT	529	T vs C	170	60	2
		316	5'-ATGAGGTGAGGATCGTGGAG					
Apbb1	BC048395	317	5'-GCTCCGCTACCAGAAGTGTC	499	C vs T	255	60	2
		318	5'-CCATGAGACCAGTCCTCTCC					
Smpd1	NM_011421	319	5'-CAGGGCTCGAGAAACCTATG	482	T vs C	246	60	2
		320	5'-CCAGGGGTAATCCAGATCCT					
Gvin1	BC030868	321	5'-TGGACCAAAATCACAAAGCA	535	G vs T	390	59	1.5
		322	5'-TCCAGGAACTCAGACCCAAC					
Trim66	NM_181853	327	5'-CCTCTGGCCAGGCATTATTA	450	C vs T	80	60	2
		328	5'-TCCTTCCAGCTCTCACACCT					
Ampd3	NM_009667	329	5'-CTCCCAATTTTGGTTGCACT	626	G vs A	123	59	1.5
		330	5'-CACAGGACATCAGAGCCAGA					
Nox4	NM_015760	365	5'-GGTTAATCTGCGAGCCAAAG	492	T vs C	270	60	2
		366	5'-AGGGGGAGCAGATTTTCCTA					
Tyr	NM_011661	367	5'-GGAACAGCGATGGGAAACTA	460	T vs A	101	60	2
		368	5'-ACAAGGCACTGGGTTGATGT					
Trim21	NM_009277	371	5'-CGGAAGAAGATGCTGAGGAC	505	G vs A	132	60	2
		372	5'-TGAAGAAAGGTCGCAGAGGT					
Trim6	NM_001013616	387	5'-CGGAGACAGGTGAGGTTTGT	420	C vs T	212	60	2
		388	5'-GAAGGTGTAGATGGGCAAGC					
Trim34 (1)	NM_030684	389	5'-CCCTTCTGTCATGCTTGGAT	583	G vs T	77	60	2

		390	5'-TGCAAGGAAGACGACTAGCA					
Trim34 (2)	NM_030684	391	5'-AGCAGGAGAAGGTGGAGACA	633	T vs C	60	60	2
		392	5'-TGTAAACTCCCAGGGTCCAG					
Trim12	NM_023835	393	5'-AAGAAAAGCCAGGGACTGGT	254	size difference		60	2
		394	5'-CCTTAGCTATGGCCACCAAA					
Ric3	NM_001038624	413	5'-GCCTTATTCTTTGGGCCTTC	464	C vs T	209	60	2
		414	5'-ATGCACAGCCTTTCAGCTTT					
Grm5	NM_001081414	417	5'-GGAAGTGCACAGTCCAGTGA	422	T vs C	107	60	2
		418	5'-TGAACTTGAATGGCCACAGA					
Mrpl17	NM_025301	419	5'-TTTGACCATGCTTACCACCA	634	G vs C	184	60	2
		420	5'-TACCCTGGCCATTCTGAAAC					
Ttc23	NM_025905	421	5'-CCACCGGACAAAAAGAAAGA	644	G vs C	327	60	2
		422	5'-GCTCCAACTTGGGAAGAGTG					
P4ha3	NM_177161	423	5'-AGTCTGGAGGCCACTGAGAA	481	CA vs TG	481	60	2
		424	5'-TCCTGGCCATCCTCTTATTG					
Sv2b	NM_153579	433	5'-AGCTGTATCCCACCAACCAG	456	C vs T	158	60	2
		434	5'-CCTGGGATGAAGAGACCTGA					
Rgma	BC059072	458	5'-GCTGACTGCTGCACACCTAC	283	G vs A	175	60	2
		459	5'-GCGTAGCACTGGGTAGGAAG					
Slco2b1	NM_175316	468	5'-GGAGATTTCCAAATGCAGGA	507	A vs G	220	59	1.5
		469	5'-CTGGGTCAGATGAGCCTAGC					
Mtap6	NM_001048167	470	5'-GAACCGAGGTCCTGTAACCA	609	G vs A	171	59	1.5
		471	5'-CTGGGGGCTGTTGTATCTGT					
Man2a2	NM_172903	472	5'-CTACGGACATGGTCCCTGAT	575	G vs T	124	60	2
		473	5'-GGCCAGTTCCTTGTTCACAT					
Il16	NM_010551	474	5'-GCTGGAAGCTCCAGTAATCG	601	C vs T	78	60	2
		475	5'-CAGGAGTGTGACAGGAGCAA					
Vps33b	NM_178070	480	5'-ATCGGTCCCTGAAAACACAG	627	G vs A	148	60	2
		481	5'-ACTGGCCAGGAAAACATCAG					

Genomic DNA primers

**Gene**		**Primer No**	**Primer seq**	**Primer location**		
				78,984,845	60	2
Agc1	NM_007424	255	5'-GGTCACTGTTACCGCCACTT	78,985,185		
		437	5'-AGAAAATGTGGGACCGTTTG	56,403,747	60	2
p	NM_021879	456	5'-GCCTGTCCTTTCTTCATTCC	56,404,332		
		266	5'-GTGGATTTGCACCATTTCTT	97,292,393	60	2
Thrsp	NM_009381	293	5'-CCGAAGAAGACAGGATCTCG	97,292,071		
		455	5'-ACTGGGTGAGAAGGCTGAGA	100,166,774	60	2

P4ha3	NM_177161	439	5'-TCAGGCGTCTCCTTCTCTGT	100,172,239		
		440	5'-TCTGTGGCGATGGTAAAACA			

Bisulfite PCR primers

**Gene**		**Primer No**	**Primer sequence**	**Primer location**	**PCR prod. (bp)**	T_A** (°C)
H47	NM_024439	493	5'-TTTTTGAGATAGTGGATTAGAAGAAAG	65,958,232	288	51
		494	5'-CACAAAAAAAATATAACCAAAAAAC	65,958,494		
Agc1	NM_007424	495	5'-TTATGTGTGGATTTTTAGTAGTAAATATA	78,921,739	188	51
		496	5'-TTAAAACACAAACCTACAAAAACAC	78,921,902		
Thrsp	NM_009381	462	5'-TTGTTTTGTTATTTTTTGGTATTTT	97,292,252	546	53
		463	5'-ACTCCAATAATTAAACTTTAACATCCTATC	97,292,768		
p	NM_021879	464	5'-TAATTTTTGATATTTTTGGGAGGTT	56,108,071	336	53
		465	5'-AATTTAAACAATAATTCTCAACTTCACTAC	56,108,377		
P4ha3	NM_177161	460	5'-GTTTTAGTAAGGGTTTGTGGGAAAG	100,162,706	449	53
		461	5'-ACCCAAACAAAACACAAAAACCTAA	100,163,130		

*, SNP distance (bp) from upstream primer

T_A **, annealing temperature. See Materials and Methods or the legend to Fig. S4 for other details of PCR.

[a] and [b]: See Table S1 legend.

Note: All sequences are based on NCBI mouse genome Build 36 (April 2006, strain C57BL/6J)
