# Supplementary material for: Monoallelic Expression of Multiple Genes in the CNS
Source: PLoS One. 2007 Dec 12;2(12):e1293. doi: 10.1371/journal.pone.0001293 (PMC2100171; doi:10.1371/journal.pone.0001293)
Supplement: Table S2 — Bi-allelic expression of genes on mouse Chr 7. The list includes 36 genes analyzed by RT-PCR of clonal hybrid neural stem cell lines (0.09 MB PDF) [file pone.0001293.s006.pdf]

Table S2. Bi-allelic expression of genes on mouse Chr 7.
The genes listed showed bi-allelic expression in all of the NSC clonal lines that gave a detectable signal. Primers and allelic differences are shown in Table S1. Genes selected because of proximity to *OlfR* genes are marked by an asterisk.

| Gene i.d. | Gene symbol | Transcription start site | # Cell lines assayed |
|---|---|---|---|
| AK082155 | --- | 83426443 | 6 |
| AK158571 | --- | 51874037 | 6 |
| BC066148 | --- | 92749397 | 5 |
| NM_029335 | --- | 83671011 | 5 |
| AK162999 | *Akap13* | 75485345 | 6 |
| NM_009667 | *Ampd3* | 110563780 | 4 |
| NM_009682 | *Ap3s2* | 79794119 | 6 |
| BC048395 | *Apbb1* | 105432308 | 6 |
| NM_175105 | *Aqp11* | 97613439 | 6 |
| NP_031628 | *Capn5* | 97996757 | 6 |
| AK129255 | *Chsy1* | 65988464 | 6 |
| NM_001005232 | *Dbx1* | 49499520 | 4 |
| NM_172904 | *Fsd2* | 81440457 | 6 |
| NM_008055 | *Fzd4* | 89279557 | 6 |
| NM_201352 | *Gdpd5* | 99255631 | 6 |
| NM_001081414 | **Grm5 (2)* | 87477812 | 6 |
| BC030868 | *Gvin1* | 105872797 | 6 |
| NM_010551 | *Il16* | 83519249 | 6 |
| AK075909 | *Lrrc28* | 67387919 | 6 |
| BC072664 | *Lrrk1* | 66144396 | 6 |
| BC075646 | *Luzp2* | 54703633 | 3 |
| NM_172903 | *Man2a2* | 80233965 | 6 |
| NM_025301 | **Mrpl17* | 105684908 | 6 |
| NM_001048167 | *Mtap6* | 99141530 | 6 |
| NM_015760 | **Nox4* | 87122559 | 4 |
| NM-183168 | *P2ry2* | 100870659 | 4 |
| AK159012 | *Pde2a* | 101295730 | 6 |
| BC024756 | *Plekhb1* | 100531018 | 6 |
| BC059072 | *Rgma* | 73264739 | 6 |
| NM_001038624 | **Ric3* | 108874501 | 6 |
| NM_175316 | *Slco2b1* | 99531887 | 6 |
| NM_011421 | *Smpd1* | 105428236 | 6 |
| NM_153579 | *Sv2b* | 75080208 | 4 |
| AB057760 | *Sytl2* | 90223891 | 6 |
| NM_026795 | *Tm2d3* | 65572408 | 6 |
| NM_145375 | *Tm6sf1* | 81732643 | 6 |

| | | | |
|---|---|---|---|
| NM_181853 | *Trim66* | 109284915 | 6 |
| NM_025905 | *Ttc23* | 67520962 | 6 |
| NM_001013616 | **Trim6* | 104106136 | 3 |
| NM_009277 | **Trim21* | 102439262 | 6 |
| NM_030684 | **Trim34 (1)* | 104118278 | 6 |
| NM_030684 | **Trim34 (2)* | 104203369 | 6 |
| NM_011661 | **Tyr* | 87368293 | 3 |
| NM_178070 | *Vps33b* | 80143457 | 6 |
| NM_178707 | *Zfp592* | 80867225 | 6 |
