# Supplementary material for: Monoallelic Expression of Multiple Genes in the CNS
Source: PLoS One. 2007 Dec 12;2(12):e1293. doi: 10.1371/journal.pone.0001293 (PMC2100171; doi:10.1371/journal.pone.0001293)
Supplement: Text S1 — Automated peak selection. The computational method used to analyze the peaks obtained by microarray hybridization is described. (0.02 MB DOC) [file pone.0001293.s007.doc]

**Text S1. Automated peak selection.**

The results of each microarray hybridization were listed as log_2_ ratios of signal intensity for each data point centered about 0. Peaks were detected by Signal Map peak finding software (Nimblegen, Madison, WI) and the statistical program JMP (SAS, Cary, NC), as follows: 1) Using a “supervised learning” approach, we optimized the threshold and window size to minimize false negatives while including the peaks in the control region of *Snrpn* as well as the peaks for the four genes identified in this study as monoallelically expressed. For each slide, the threshold level was set at the 75^th^ percentile of all data points (range of log_2_ ratios, 0.36-0.52). Peaks were defined by the default Signal Map setting (at least 4 adjacent probes showing signal intensities above the threshold level), with a sliding window of 300 bp (McrBC-treated DNA *vs*. control) or 1000 bp (RE mix-treated DNA *vs*. control) in increments of 100 bp.

For each peak the raw signal intensity was defined as the maximum log_2_ ratio within the peak. To facilitate comparison between slides, values in the top 10 percentile were equated to 1000, and the remaining peaks were linearly reset on a scale of 0 to 999. For all slides analyzed, a cutoff score of 300 was used, representing a minimum 1.9 fold increase above the control value.

For each mouse, coincidence of peaks between the two hybridizations was determined in a two-step process. We first selected peaks computationally that were within 1500 base pairs of one another. Of these we selected the subset of peaks that were within the same Csp6I fragment in both hybridizations, as determined visually by use of the UCSC genome browser.
