# Supplementary material for: Monoallelic Expression of Multiple Genes in the CNS
Source: PLoS One. 2007 Dec 12;2(12):e1293. doi: 10.1371/journal.pone.0001293 (PMC2100171; doi:10.1371/journal.pone.0001293)
Supplement: Text S2 — Isolation of NSCs and clonal cell lines (0.04 MB DOC) [file pone.0001293.s008.doc]

**Text S2. Isolation of NSCs and clonal cell lines.**

**Derivation of neural stem cell lines**

We used 6-to-8 week old female mice from reciprocal crosses of C57BL6 (B6) and Japanese Fancy Mouse 1 (JF1). Two cell lines were generated; NSC2 from the cross JF1♀ x B6♂, and NSC3 from the cross B6♀ x JF1♂. We used slightly modified published procedures throughout (Palmer et al. 1999; Shi et al. 2004). For each cell line, four mouse forebrains were collected and weighed, then transferred to a small amount of PBS/PSF (PBS, Dubecco’s Phosphate Buffered Saline solution Ca++/Mg++ free (Irvine Sci.#9240); PSF, antibiotic-antimycotic solution (Invitrogen #15240-062)). Each forebrain was gently teased apart, and the minced slices were then combined in a 50 ml conical tube, and washed with PBS/PSF by trituration with a plastic transfer pipet. After the tissue settled for several minutes, the tube was centrifuged for 5 s, and the supernatant was slowly removed.

Papain (Worthington, #3126) was pre-warmed to room temperature and added at a final concentration of 2.5 U/ml to pre-warmed DMEM F12 (Omega Sci., DM25) together with Dispase II (Roche,#165859) (final concentration 2mg/ml), and DNAse I (Worthington, #2139) (final concentration 250 U/ml). The entire digestion solution was filter sterilized (0.2 µm filter), then added immediately to minced tissue for 30 min at 37ºC (10 ml solution/g tissue). The minces were triturated every 10 min, and centrifuged at 2000 rpm for 3 min at the end of digestion. Some minced tissue was still visible following digestion.

The digests were washed 3X with DMEM F12 (Omega Sci., DM25) + 10% heat inactivated FBS (DMEM-FBS) (80 to 100 ml/g tissue), then the cells were collected by centrifugation at 2,000 rpm for 3 min. The washed cells were suspended in 11 ml of DMEM-FBS, followed by addition of 11 ml of Percoll (Amersham, #17-0891-01) in PBS. The cells were split into two aliquots in autoclaved polyallomer ultracentrifuge tubes, and centrifuged in an SW 41 rotor at 12,700 rpm for 30 min at room temperature to form a gradient containing 3 layers.

For each tube, the top (largely lipid) layer, and the top half of the second layer were removed by aspiration. A Pasteur pipet and pipetman were then used to transfer the bottom portion of the second layer containing the NSCs to a fresh 50 ml conical tube, minimizing disturbance of the third (bottom) layer containing red blood cells. The entire procedure was carried out slowly, with pipets held vertically, so as to avoid drawing cells from the walls of the tubes. The cells were washed three times with 50 ml PBS/PSF to remove residual Percoll, then collected by centrifugation at 2,000 rpm for 3 min. The two aliquots were then combined, and washed once more with DMEM F12 supplemented with N2 (Invitrogen, #17502-048).

The cells were resuspended in 1 ml of DMEM F12, supplemented with 1 mM L-glutamine (Omega Sci., GS60), N2, recombinant human epidermal growth factor (EGF) (20ng/ml) (Peprotech, #100-15), recombinant human fibroblast growth factor (FGF-2) (20ng/ml) (Peprotech, # 100-18 B), heparin (5 µg/ml) (Sigma, # H 3149) and PSF (termed culture medium). Cells were triturated with a fire polished Pasteur pipet to separate clumps. The cells were then counted, and plated onto 1-2 60 mm x 15 mm tissue culture dishes. There should be 5 X 10^5^ to 2 X 10^6^ NSCs (round bright cells), and an equal number of red blood cells (small dim cells).
 Cells were cultured in culture medium (5 ml media/ dish) in 5 % CO_2_, 10 % O_2_ and 85 % N_2_ for the first 1-2 months. In some cases conditioned medium was used: It was collected from confluent NSC cells, filtered (0.2 µm), and stored at 4ºC for up to 1 week, or at – 20ºC or -70ºC for longer periods

At days 2, 3 and 4, EGF (20 ng/ml), FGF 2 (20 ng/ml), and heparin (5 µg/ml) were added to each dish. If the cells did not appear to be growing well, higher concentrations of growth factors were used. For cells growing quickly, ½ medium was replaced starting at day 4-5, and continued for ~4 weeks; thereafter, medium was changed completely every 2-3 days. For sparse cells the entire process was carried out on a slower schedule, with growth factors and heparin added every 2-3 days, but medium left unchanged for the first 1-3 weeks.

Cells were monitored by microscopy. At about the time that contaminating cells (mostly fibroblasts) and debris were no longer present, stem cells began to form colonies having a filigreed appearance; dishes containing fewer cells occasionally formed neurospheres.

Cells were transferred when confluent. To transfer attached cells, trypsin-EDTA (Invitrogen # 15400-054) was added to cover each dish (0.5ml /60 mm dish) then aspirated immediately. The cells were shaken off by tapping the side of the dish, then 1 ml of culture medium was added, and the cells were transferred to a 100 mm x15mm culture dish (10 ml culture medium/dish). Cells were then serially passaged at confluence, with trypsinization carried out as described above (1 ml trypsin/100mm dish). Cells were frozen overnight -70 ºC in freezing medium (culture media + 10 % vol/vol Dimethylsulfoxide(DMSO) (Sigma, #D 2650), then stored in liquid N_2_.

**Cloning neural stem cells**

*Preparation of feeder layers and conditioned medium*

Culture medium (400 µl) was added to culture plate inserts (Millipore, # PICMO1250 placed in wells of 24-well plates. Additional medium (600 µl) was then added beneath each insert. Cells were then seeded onto each insert (1x10^5^ cells/ insert). Every 2-3 days ½ media was replaced in both the wells and inserts. When the cells began forming neurospheres, inserts were either a) removed to provide feeder cells (see below) and replaced with fresh seeded inserts, or b) used to collect conditioned medium. In the latter case, medium was collected from both the inserts and wells, and pooled, until such time as the neurospheres began to become necrotic (dark in color) and/or the culture medium turned yellow. Conditioned medium was filtered and stored as described above.

*Single cell cloning*

One ml of culture medium was added to trypsinized late log phase NSCs (100 mm plate), and the cells were dissociated by trituration with a fire polished Pasteur pipet. Cells were counted, diluted to ~1 to 2 cells/10 µl in culture medium, and distributed to several 24-well plates containing 600 µl medium. The cells were allowed to settle for 4 hrs, then wells containing single cells were marked for subsequent addition of inserts containing mature feeder cells (see above). Approximately 20-30% of the wells contained individual NSCs, identified microscopically as round cells with nearly invisible nuclei that form processes only after several rounds of cell division. Cells were monitored and treated daily until colonies formed, as follows:

Day 1, Plate single cells.

Day 2, Change 200 µl of culture media in the insert. If desired, additional amounts of the growth factors EGF and FGF-2 may be added (10 µl of 1:100 dilution of stock to each well).

Day 3, Replace 200 µl medium in each insert, and 300 µl medium in each well. In some wells, cells should start to divide.

Day 4, Some cells should be dividing and have processes. Additional growth factors may be added as above.

Day 5, Change ½ medium in each insert and well, as above.

Day 6, In some wells, small NSC colonies start to form (circle of bright cells with small processes). Add additional growth factors as above.

Day 7, Change ½ medium in each insert and well, as above.

Day 10, Replace inserts in each well with fresh inserts containing feeder cells.

We continued the regimen above until cells appeared confluent. They were then trypsinized, and serially passaged as described above, progressing from 6 well plates, to 60 mm plates, then to100 mm plates. Medium was replaced every 2-3 days, with a gradual shift from conditioned medium to regular medium.

**Karyotype analysis**

Cells were sub-cultured into 60 mm dishes at three different concentrations, to optimize the number of dividing cells. Karyotyping was performed as described (Robertson 1987).

**References**

Palmer TD, Markakis EA, Willhoite AR, Safar F, Gage FH (1999) Fibroblast growth factor-2 activates a latent neurogenic program in neural stem cells from diverse regions of the adult CNS. J Neurosci 19(19): 8487-8497.

Robertson EJ (1987) Embryo-derived stem cell lines. In: Robertson EJ, editor. Teratocarcinomas and embryonic stem cells: a practical approach. Oxford: IRL Press. pp. 71-112.

Shi Y, Chichung Lie D, Taupin P, Nakashima K, Ray J et al. (2004) Expression and function of orphan nuclear receptor TLX in adult neural stem cells. Nature 427(6969): 78-83.
